# Integrated analysis identifies TfR1 as a prognostic biomarker which correlates with immune infiltration in breast cancer

**DOI:** 10.18632/aging.203512

**Published:** 2021-09-13

**Authors:** Fei Chen, Yumei Fan, Jiajie Hou, Bing Liu, Bo Zhang, Yanan Shang, Yanzhong Chang, Pengxiu Cao, Ke Tan

**Affiliations:** 1 Key Laboratory of Animal Physiology, Biochemistry and Molecular Biology of Hebei Province, Key Laboratory of Molecular and Cellular Biology of Ministry of Education, College of Life Sciences, Hebei Normal University, Shijiazhuang, Hebei 050024, China

**Keywords:** TfR1, breast cancer, prognosis, immune infiltration, iron

## Abstract

Breast cancer (BC) is the most common malignancy with high morbidity and mortality in females worldwide. Emerging evidence indicates that transferrin receptor 1 (TfR1) plays vital roles in regulating cellular iron import. However, the distinct role of TfR1 in BC remains elusive. TfR1 expression was investigated using the TCGA, GEO, TIMER, UALCAN and Oncomine databases. The prognostic potential of TfR1 was evaluated by Kaplan-Meier (KM) plotter and univariate and multivariate Cox regression analyses. Moreover, Gene Ontology (GO), Kyoto Encyclopedia of Genes and Genomes (KEGG) and gene set enrichment analysis (GSEA) were used to explore the molecular mechanism of TfR1. The potential link between TfR1 expression and infiltrating abundances of immune cells was examined through the TIMER and CIBERSORT algorithm. The expression of TfR1 was dramatically upregulated in BC tissues. Increased TfR1 expression and decreased methylation levels of TfR1 were strongly correlated with multiple clinicopathological parameters. Elevated TfR1 expression was associated with a poor survival rate in BC patients. The nomogram model further confirmed that TfR1 could act as an independent prognostic biomarker in BC. The results of GO, KEGG and GSEA revealed that TfR1 was closely correlated with multiple signaling pathways and immune responses. Additionally, TfR1 was positively associated with the infiltration abundances of six major immune cells, including CD4+ T cells, CD8+ T cells, B cells, neutrophils, macrophages, and dendritic cells in BC. Interestingly, TfR1 influenced prognosis partially through immune infiltration. These comprehensive bioinformatics analyses suggest that TfR1 is a new independent prognostic biomarker and a potential target for immunotherapy in BC.

## INTRODUCTION

Among malignancies, breast cancer (BC) is the leading cause of cancer death and has the highest morbidity rate in women worldwide [1]. With the development of treatment, the survival rate of BC patients has been significantly improved [2, 3]. However, a large number of patients still die from BC because of metastasis and/or chemoradiotherapeutic resistance [2–4]. Therefore, it is crucial to investigate the mechanisms that lead to the incidence of BC metastasis and chemoresistance and identify some prognosis-related factors of BC.

Iron is an essential trace element indispensable for many biological processes, such as DNA replication, erythropoiesis, cell cycle, oxidative metabolism, and mitochondrial respiratory and cellular immune responses [5, 6]. Most cancer cells need a relatively high concentration of cellular iron to ensure their rapid proliferation and growth [7, 8]. Systemic and intracellular iron homeostasis is tightly maintained in a sophisticated system and mediated by iron uptake, storage, utilization and export [5–8]. Alterations in the expression of iron homeostasis-associated genes have been identified as potential prognostic biomarkers or therapeutic targets in some cancers [9, 10].

The major protein essential for the uptake of iron is transferrin receptor 1 (TfR1), encoded by *TFRC,* which is found primarily as a homodimer [11]. TfR1 is a type II transmembrane glycoprotein that is endocytosed from the cell membrane after binding iron with transferrin (Tf), a serum iron carrier protein [12]. In contrast, TfR2 is expressed in certain tissues, such as in the liver and duodenum, as well as in erythrocytes, and plays a minor role in iron uptake. Given the important role of TfR1, its dysregulation may be associated with persistent iron stimulation and cancer development and progression [13]. Consistent with this speculation, aberrant levels of TfR1 have been studied in several types of cancer, including myeloma and lung, liver, colon, brain and ovarian cancers [13, 14]. Furthermore, TfR1 was found to affect many aspects of tumorigenesis, such as cancer cell proliferation, migration, invasion, metastasis and apoptosis [14, 15]. Regrettably, the expression profile and prognostic role of TfR1 in BC are still not unknown. The connection between TfR1 and the immune response also remains to be explored. Thus, the aims of this study were to compare the expression of TfR1 in BC and normal breast tissues, to explore the prognostic significance of TfR1 expression, to analyze TfR1-related signaling pathways and to reveal the link between TfR1 and the infiltration abundances of various immune cells in BC by pooling currently available data online. In summary, our results provide new insights into the feasible function of TfR1 in cancer immune regulation and its utilization as a potential cancer biomarker.

## RESULTS

### The mRNA and protein levels of TfR1 in different types of cancer

**Figure 1:**
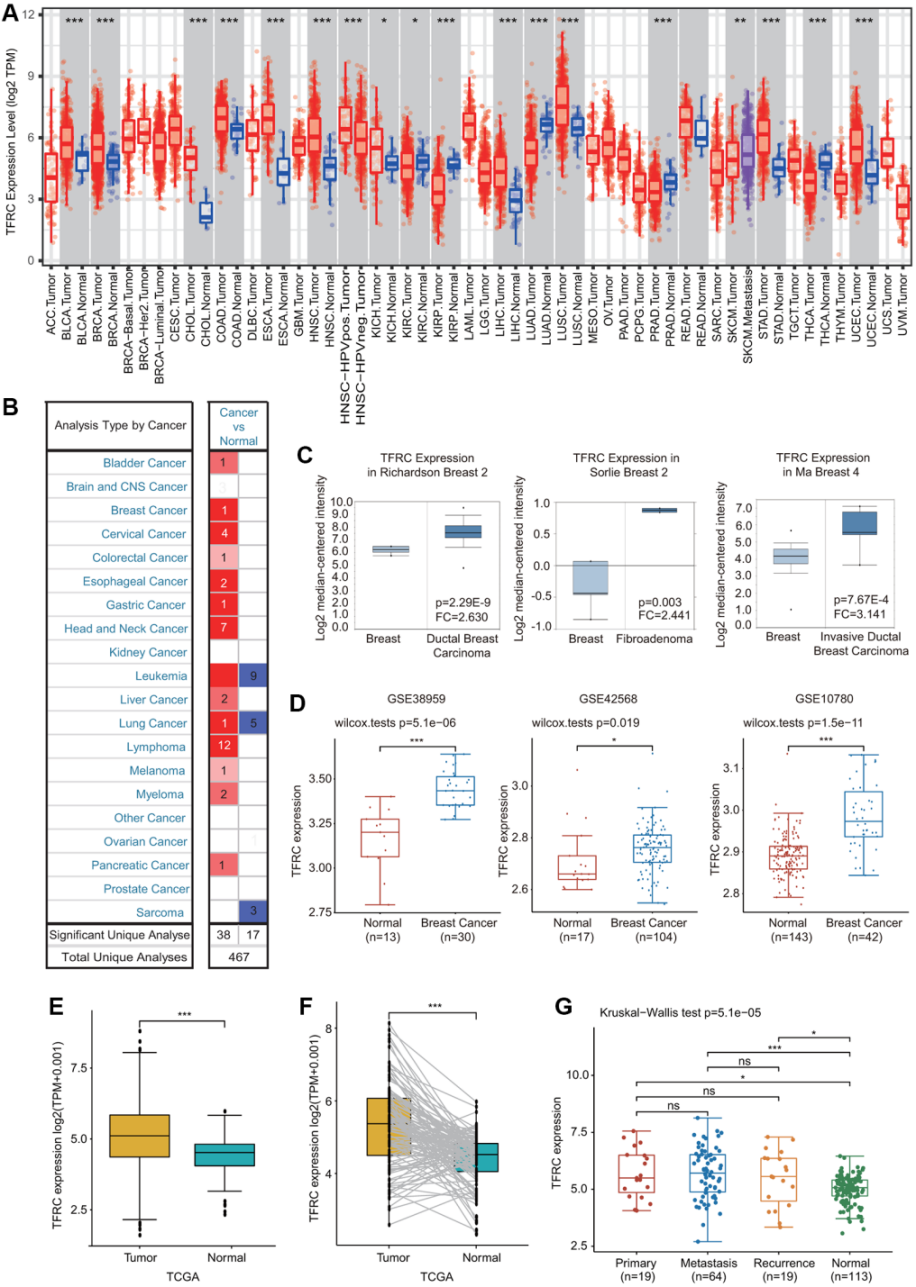
**Expression of TfR1 in BC.** (**A**) The expression of TfR1 in various cancers from the TIMER database. (**B**) The change in TfR1 expression in common cancers was obtained using the Oncomine database. (**C**) TfR1 is overexpressed in BC tissues in the Oncomine database. (**D**) TfR1 was overexpressed in BC tissues (GSE38959, *n* = 30; GSE42568, *n* = 104; GSE10780, *n* = 42) compared with adjacent normal tissues (GSE38959, *n* = 13; GSE42568, *n* = 17; GSE10780, *n* = 143) in the different GEO datasets. (**E**) TfR1 expression is elevated in BC tissues compared with noncancerous adjacent tissues from the TCGA database. (**F**) TfR1 expression in 112 matched BC tissues and adjacent normal tissues in the TCGA database was investigated. (**G**) TfR1 expression in primary cancer, metastasis and recurrence in BC patients. ^*^< 0.05, ^**^< 0.01, ^***^< 0.001.

First, the expression of TfR1 in common human cancers was assessed using the TIMER database. Upregulated TfR1 expression was observed in BLCA, CHOL, COAD, ESCA, HNSC, KICH, LUSC, LIHC, STAD and UCEC tissues, whereas downregulated TfR1 expression was observed in LUAD, KIRP, KIRC, PRAD and THCA tissues (Figure 1A). Consistently, elevated TfR1 expression was shown in BC tissues compared with normal breast tissues through the Oncomine online database (Figure 1B, 1C and Supplementary Figure 1). Additionally, three GEO datasets (GSE42568, GSE38959 and GSE10780) were used to further confirm TfR1 expression in BC. The mRNA level of TfR1 was strongly increased in BC in all three GEO datasets (Figure 1D). TfR1 expression was further investigated through the TCGA database, and we found that TfR1 expression was markedly upregulated in BC tissues (Figure 1E). An increase in TfR1 expression in BC was observed when 112 paired BC and normal breast tissues were analyzed (Figure 1F). Furthermore, TfR1 expression was also upregulated in different BC patients, including those with primary cancer, metastasis and recurrence (Figure 1G).

**Figure 2:**
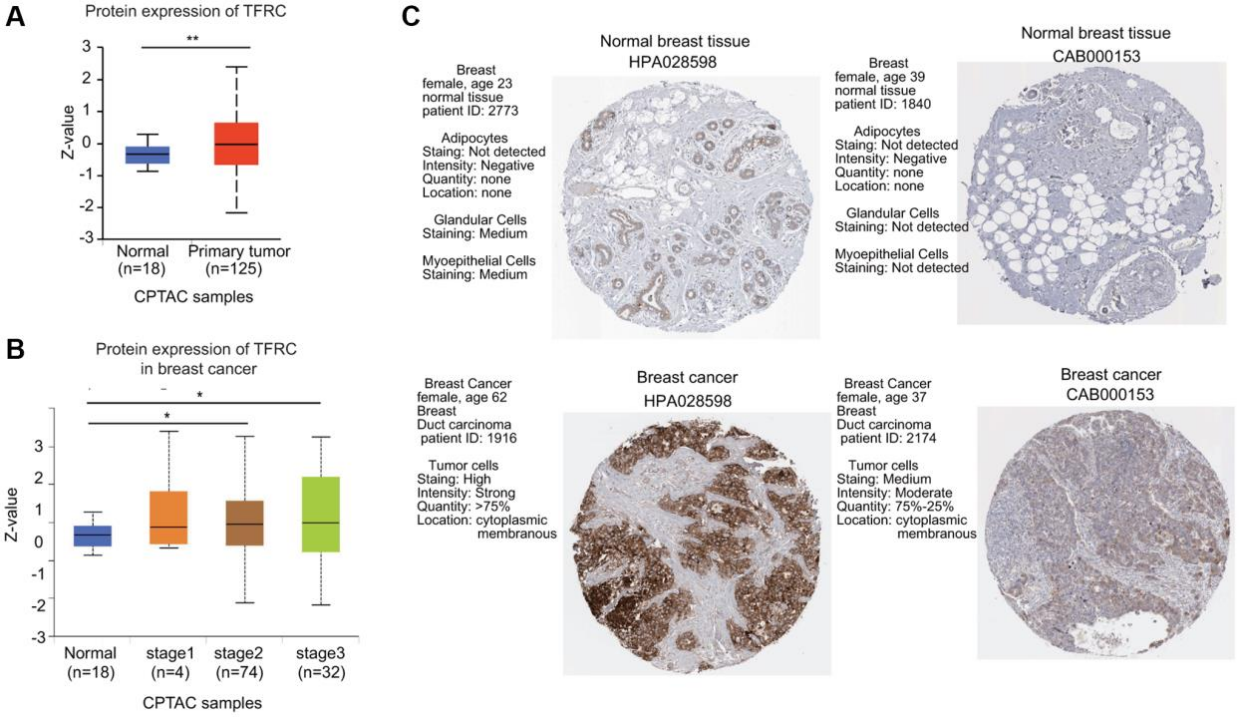
**The protein expression level of TfR1 in BC.** (**A**) TfR1 expression at the protein level in BC tissues (*n* = 125) and normal breast tissues (*n* = 18) through UALCAN database. (**B**) TfR1 protein expression was investigated in patients with different stages of BC (normal individuals, *n* = 18; stage 1, *n* = 4; stage 2, *n* = 74; and stage 3, *n* = 32). (**C**) The protein expressions of TfR1 were evaluated using the HPA database. The results of IHC staining suggested that the TfR1 protein was expressed at high levels in BC tissues. ^*^< 0.05, ^**^< 0.01.

Next, the protein levels of TfR1 in BC were evaluated using the UALCAN database. The protein expressions of TfR1 were also greatly elevated in BC tissues compared with normal adjacent samples (Figure 2A). In terms of tumor stage, increased protein levels of TfR1 were found in BC patients with stage 2 and 3 disease (Figure 2B). Moreover, IHC staining from the Human Protein Atlas (HPA) database suggested that TfR1 protein levels were highly expressed in BC tissues compared with normal breast tissues (Figure 2C).

### Relationship between TfR1 expression and different clinicopathological characteristics

**Figure 3:**
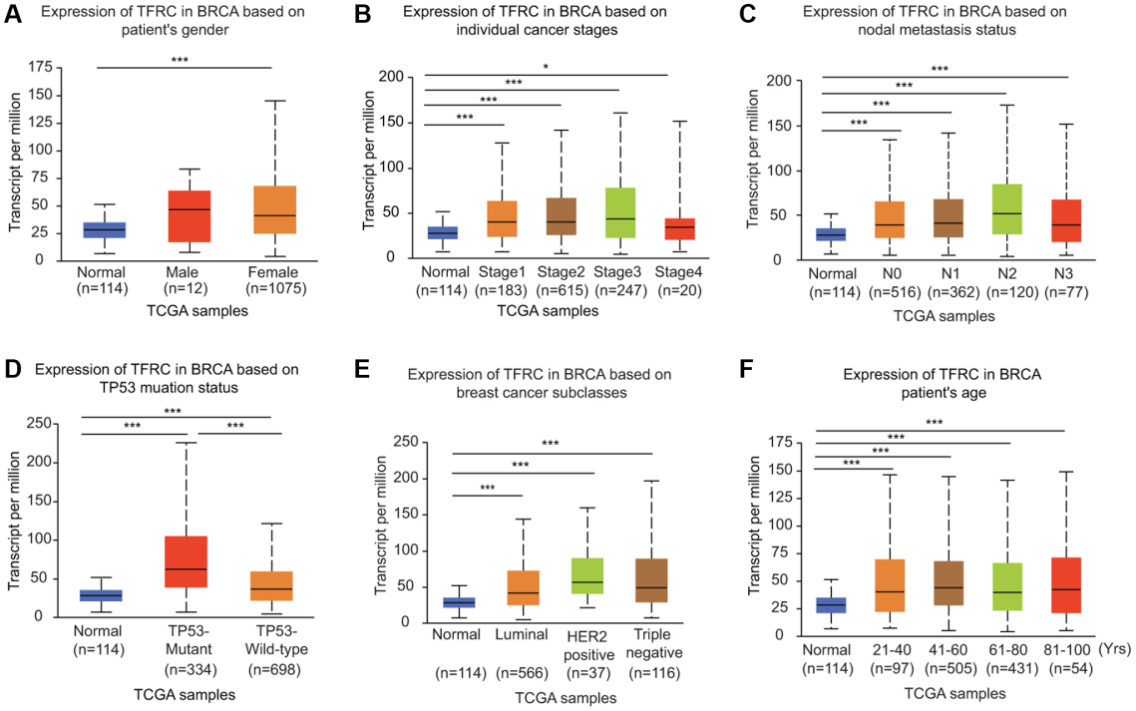
**Association of TfR1 expression with clinicopathological parameters of BC patients.** TfR1 expression was investigated in (**A**) female (*n* = 1075) and male (*n* = 12) patients, (**B**) patients with different stages of BC (normal individuals, *n* = 114; stage 1, *n* = 183; stage 2, *n* = 615; stage 3, *n* = 247; and stage 4, *n* = 20), (**C**) patients with different nodal metastasis statuses (normal individuals, *n* = 114; N0, *n* = 516; N1, *n* = 362; N2, *n* = 120; and N3, *n* = 77), (**D**) patients with different TP53 mutation statuses (normal individuals, *n* = 114; TP53-nonmutant, *n* = 698; and TP53-mutant, *n* = 334), (**E**) patients with different BC subtypes (normal individuals, *n* = 114; HER-positive, *n* = 37; luminal, *n* = 566; and triple negative, *n* = 116) and (**F**) patients with different ages (normal individuals, *n* = 114; 21–40 years, *n* = 97; 41–60 years, *n* = 505; 61–80 years, *n* = 431; and 81–100 years, *n* =54). ^*^< 0.05, ^**^< 0.01, ^***^< 0.001.

The expression of TfR1 was greatly upregulated in both female and male BC patients in the UALCAN database (Figure 3A). In addition, TfR1 was identified as a clinical stage-associated gene, and TfR1 expression was remarkably upregulated in BC patients with stage 1-4 disease (Figure 3B). According to nodal metastasis status, TfR1 expression was upregulated in BC patients classified as N0, N1, N2 and N3 (Figure 3C). Furthermore, high TfR1 levels were observed in TP53-wild-type and TP53-mutant BC patients (Figure 3D). Regarding the subtypes of BC, TfR1 expression was strongly elevated in patients with the luminal, HER2-positive and triple-negative BC (TNBC) subtypes (Figure 3E). Moreover, age was significantly related to TfR1 expression, as high TFR1 expression was observed in elderly BC patients (21–40, 41–60, 61–80 and 81–100 years) (Figure 3F). In terms of menopausal status, TfR1 expression was elevated in patients with pre-menopausal, peri-menopausal, and post-menopausal BC (Supplementary Figure 2A). TfR1 expression was also significantly upregulated in three different races (Supplementary Figure 2B).

**Figure 4:**
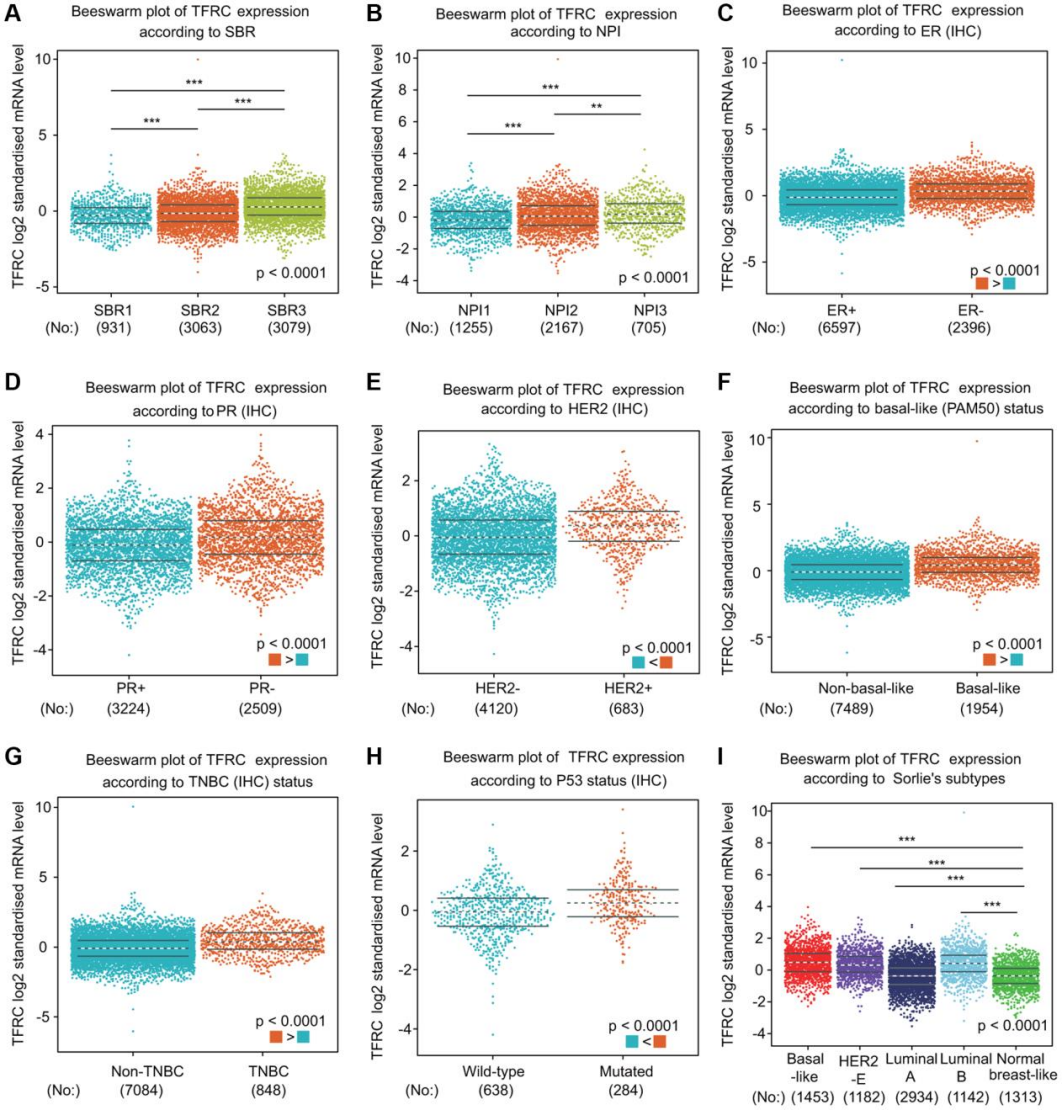
**TfR1 expression in BC patients based on different pathological parameters was assessed using bc-GenExMiner v4.5.** Box plots are shown for (**A**) SBR grade (SBR1, *n* = 931; SBR2, *n* = 3063; and SBR3, *n* = 3079), (**B**) NPI index (NPI1, *n* = 1255; NPI2, *n* = 2167; and NPI3, *n* = 705), (**C**) ER status (ER+, *n* = 6597; ER-, *n* = 2396), (**D**) PR status (PR+, *n* = 3224; PR-, *n* = 2509), (**E**) HER-2 status (HER2+, *n* = 683; HER2-, *n* = 4120), (**F**) basal-like status (non-basal-like, *n* = 7489; basal-like, *n* = 1954), (**G**) TNBC status (non-TNBC, *n* = 7084; TNBC, *n* = 848), (**H**) TP53 status (wild type, *n* = 638; mutated, *n* = 284), and (**I**) Sorlie subtypes (basal-like, *n* = 1453; luminal A, *n* = 2934; luminal B, *n* = 1142; HER2-E, *n* = 1182; and normal breast-like, *n* = 1313). ^*^< 0.05, ^**^< 0.01, ^***^< 0.001.

We further compared TfR1 expression in BC patients according to different clinical parameters through bc-GenExMiner online tool. BC patients with higher Nottingham Prognostic Index (NPI) values and more advanced Scarff-Bloom-Richardson (SBR) grades expressed higher TfR1 mRNA expression (Figure 4A, 4B). In addition, both progesterone receptor (PR) and estrogen receptor (ER) status was negatively significantly correlated with TfR1 expression (Figure 4C, 4D). In contrast, HER-2 was significantly positively associated with TfR1 expression (Figure 4E). Moreover, the expression of TfR1 was much higher in BC patients with TNBC and basic-like subtypes (Figure 4F, 4G). TfR1 expression was upregulated in TP53-mutant BC patients compared with TP53-wild-type patients (Figure 4H). In addition, TfR1 expression was higher in the HER2-enriched (HER2-E), basal-like, luminal A and luminal B subtypes than in the normal-like subtype (Figure 4I).

### Prognostic potential of TfR1 in BC patients

**Figure 5:**
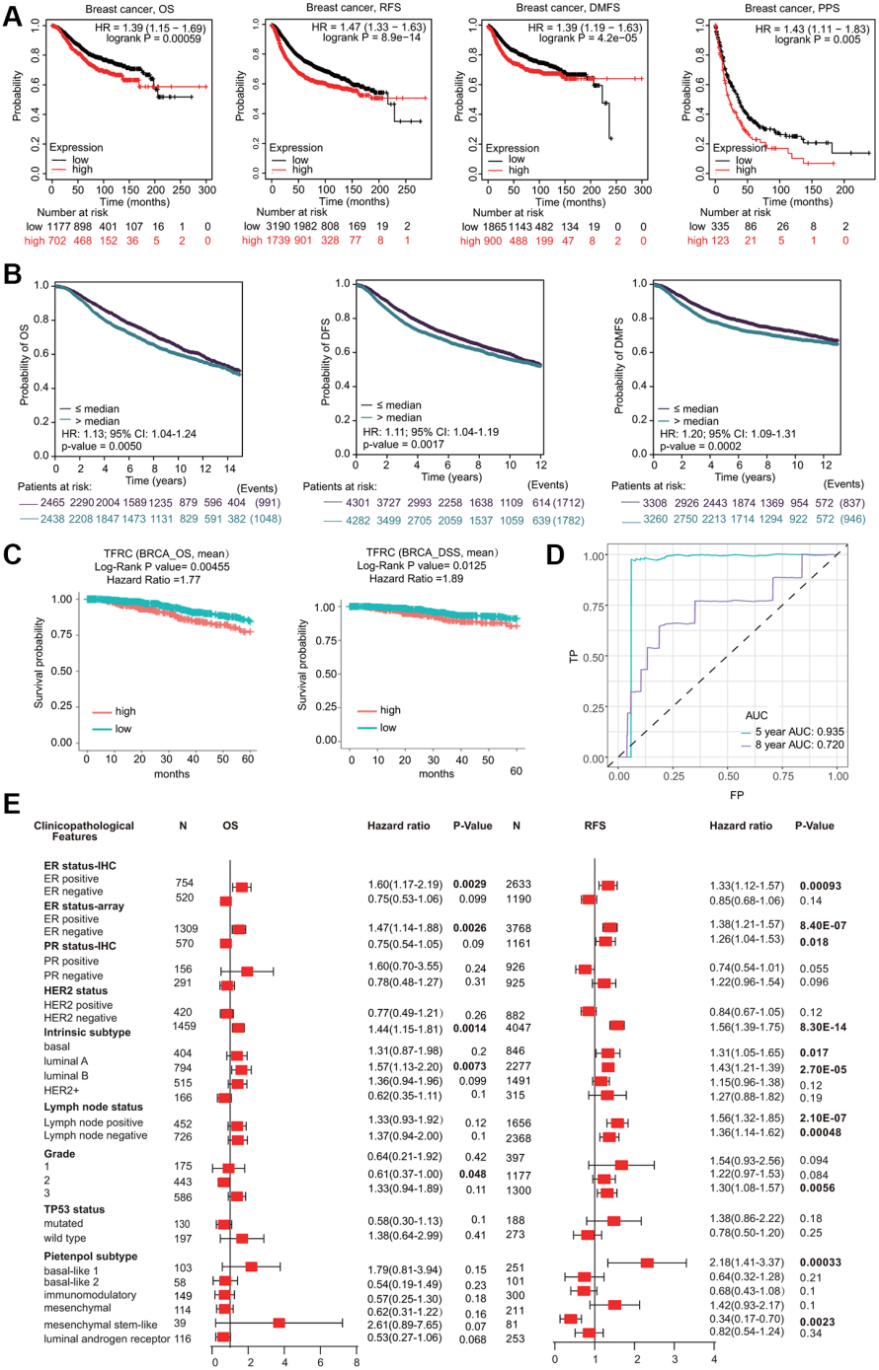
**Prognostic significant of TfR1 in BC.** (**A**) The OS, RFS, DMFS and PPS of BC cohorts were obtained from the KM plotter database. (**B**) The OS, DFS and DMFS in BC cohorts obtained through bc-GenExMiner v4.5. (**C**) The OS and DSS of BC cohorts obtained through the DiverDBv3 database. (**D**) ROC curve of TfR1 expression was shown. (**E**) Forest plots showing the associations between TfR1 expression and the clinicopathological features of patients with BC.

Interestingly, high TfR1 expression was linked with unfavorable prognosis in BC patients, including overall survival (OS), postprogression survival (PPS), recurrence-free survival (RFS) and distant metastasis-free survival (DMFS) (Figure 5A). The association between TfR1 expression and prognosis in BC patients was evaluated using the bc-GenExMiner database (Figure 5B). Elevated expression of TfR1 was linked with poor OS, distant free survival (DFS) and DMFS in BC patients (Figure 5B). Moreover, data from the DriverDBv4.5 database suggested that higher TfR1 expression was remarkably correlated with worse prognosis in terms of OS and DSS (Figure 5C). ROC (receiver operating characteristic) curve analysis was carried out to confirm the diagnostic accuracy of TfR1 for the diagnosis of BC patients. The values of AUC (area under the curve) were 0.935 and 0.72 for 5-year and 8-year survival, respectively (Figure 5D).

### Prognostic significance of TfR1 according to different clinicopathological characteristics

Kaplan-Meier (KM) plotter results demonstrated that higher TfR1 expression was significantly correlated with worse OS and RFS in BC patients with the ER-positive, HER-2-negative, and luminal A subtypes (Figure 5E and Supplementary Figure 2C). Moreover, increased TfR1 expression was linked with poor RFS in BC patients with the ER-negative, lymph node-positive, lymph node-negative, basal, and mesenchymal stem-like subtypes (Figure 5E). These results demonstrate that TfR1 expression can affect the prognosis of BC patients with diverse clinicopathological factors.

### Cox hazard regression analysis and nomogram model

**Figure 6:**
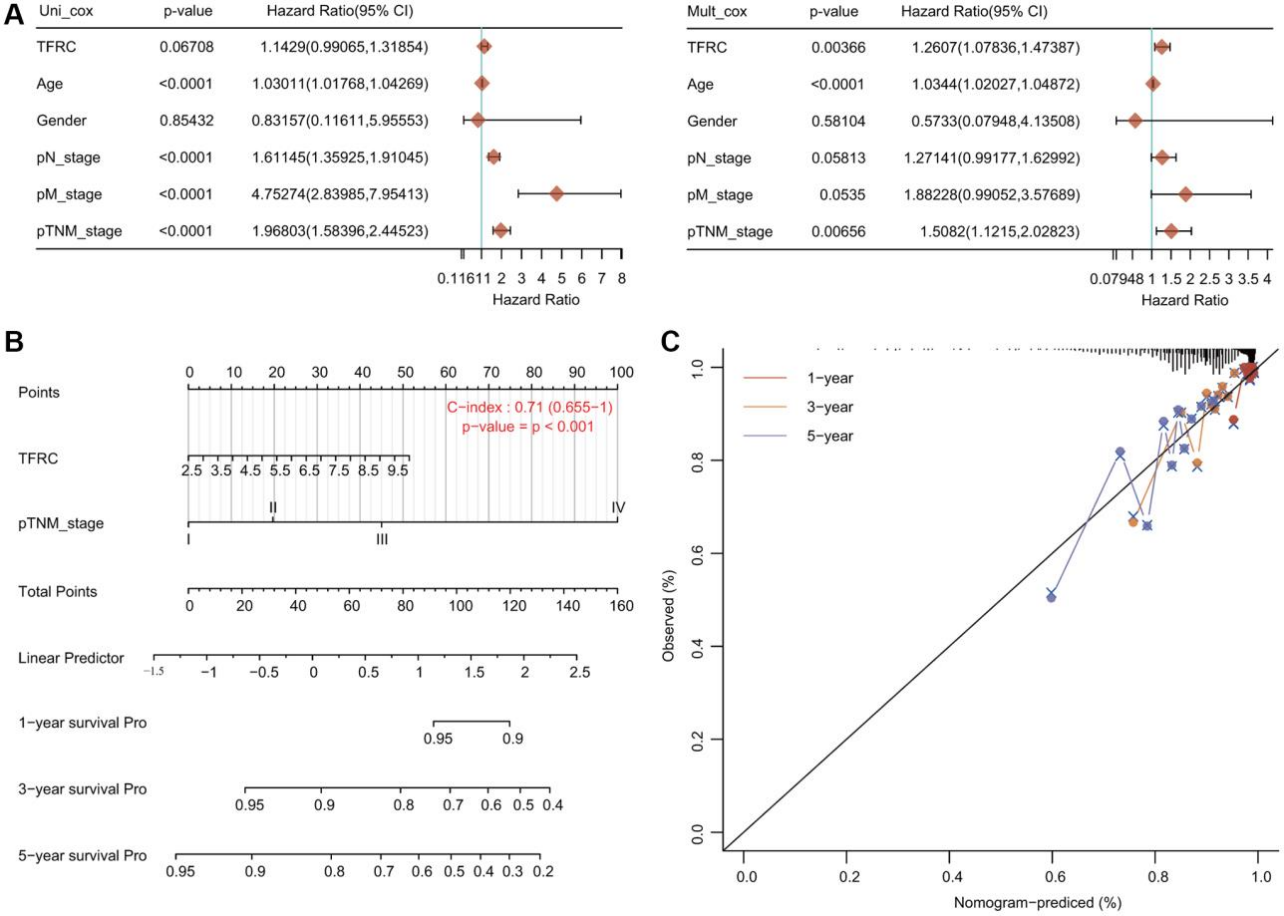
**Internal validation of TfR1 as an independent prognostic factor for BC patients.** (**A**) Univariate and multivariate Cox regression analyses determined TfR1 as an independent prognostic factor. (**B**) A prognostic nomogram integrating TfR1 expression and clinicopathologic variables was constructed to estimate OS. (**C**) Calibration plots to predict the OS of BC patients at 1, 3, and 5 years.

We carried out Cox regression analyses to further determine the potential of TfR1 as an independent prognostic factor. According to the univariate Cox regression analysis, we found that age, N stage, M stage and TNM stage were obviously correlated with the OS of BC patients (Figure 6A). According to the multivariate Cox regression analysis, TfR1 expression, age and TNM stage exhibited obvious correlations with the OS of BC patients (Figure 6A). For the luminal A subtype, the results of univariate Cox regression analysis demonstrated that age, M stage and TNM stage were strongly associated with OS (Supplementary Figure 2D). The results of multivariate Cox regression analysis suggested that age and M stage were associated with OS of the luminal A subtype (Supplementary Figure 2D).

A prognostic nomogram integrating the TfR1 expression level and clinicopathological characteristics was constructed to predict the 1-year, 3-year and 5-year OS rates of all BC patients and the luminal A subtype (Figure 6B). The concordance index (C index) of the genomic-clinicopathologic nomogram for OS prediction was 0.71 (Figure 6B). For the luminal A subtype, the C index was 0.755 (Supplementary Figure 2E). Calibration curves were plotted to compare the nomogram-predicted 1-, 3- and 5-year OS with actual OS rates (Figure 6C and Supplementary Figure 2F).

### Methylation alteration of TfR1 in BC

**Figure 7:**
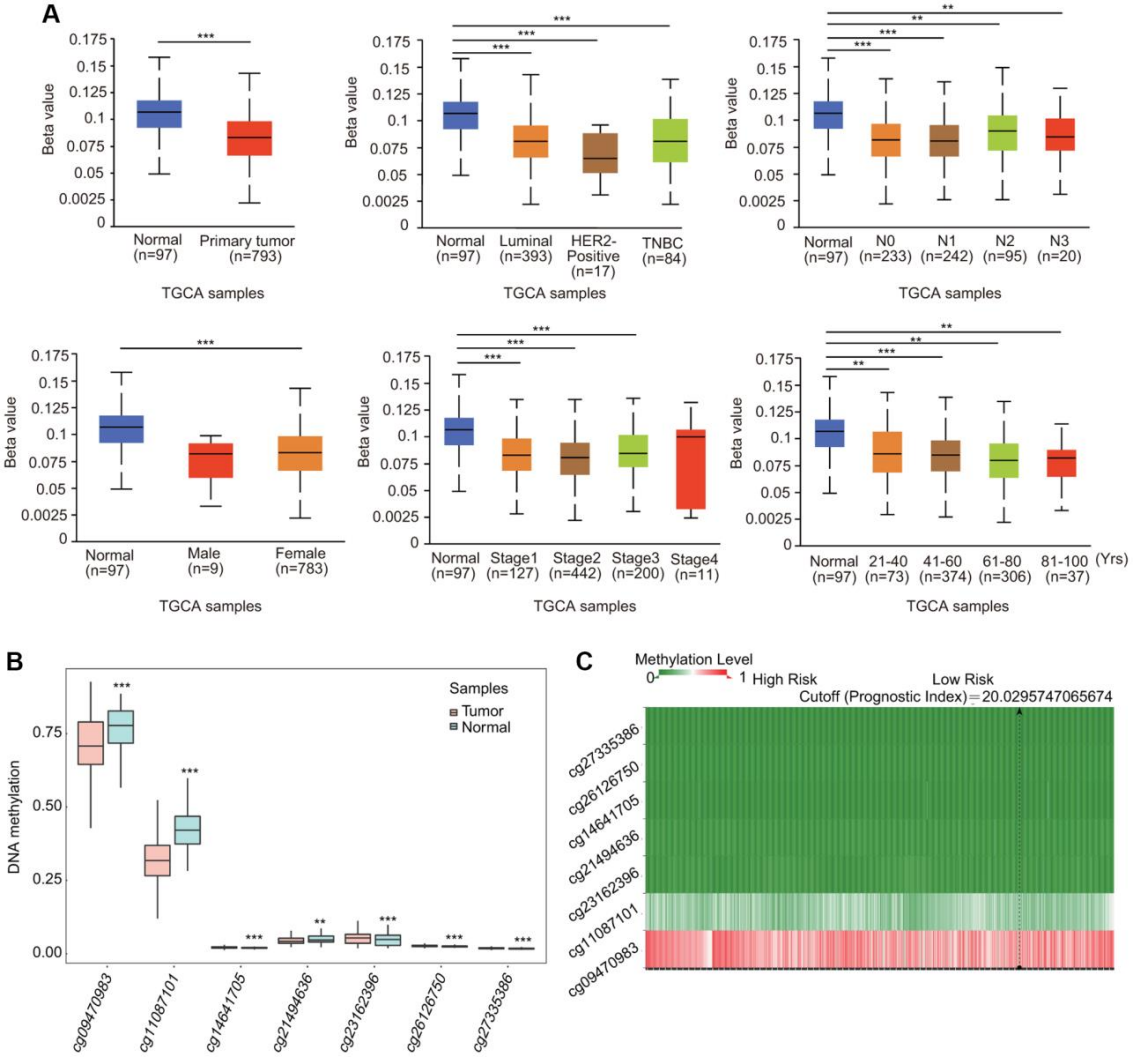
**Association of DNA methylation of TfR1 with clinicopathological parameters of BC patients.** The DNA methylation of TfR1 was investigated in (**A**) BC patients (*n* = 793) and normal individuals (*n* = 97); female (*n* = 783) and male (*n* = 9) BC patients; patients with different BC subtypes (normal individuals, *n* = 97; HER2-positive, *n* = 17; luminal, *n* = 393; and TNBC, *n* = 84); patients with different stages of BC (normal individuals, *n* = 97; stage 1, *n* = 127; stage 2, *n* = 442; stage 3, *n* = 200; and stage 4, *n* = 11); patients with different nodal metastasis statuses (normal individuals, *n* = 97; N0, *n* = 233; N1, *n* = 242; N2, *n* = 95; and N3, *n* = 20); and patients with different ages (normal individuals, *n* = 97; 21–40 years, *n* = 73; 41–60 years, *n* = 374; 61–80 years, *n* = 306; and 81–100 years, *n* = 37). (**B**) Methylation levels of TfR1 in BC through the SurvivalMeth database. (**C**) The heat map of DNA methylation of TfR1 in BC. ^*^< 0.05, ^**^< 0.01, ^***^< 0.001.

BC samples exhibited lower DNA methylation levels of TfR1 than normal samples through the UALCAN database (Figure 7A). The association of DNA methylation levels of TfR1 and clinic pathological characteristics was further investigated. DNA methylation levels of TfR1 were obviously decreased in both female and male BC patients; BC patients with stage 1, 2 and 3 disease; different subtypes (luminal, HER2-positive and TNBC); different nodal metastasis statuses (N0, N1, N2 and N3); different ages (21–40, 41–60, 61–80 and 81–100 years); different races (Caucasian, African-American, and Asian); and different menopause statuses (pre-menopausal, peri-menopausal and post-menopausal) (Figure 7A and Supplementary Figure 3A). The decreased DNA methylation level of TfR1 was further confirmed using the Survival Meth database. Three of the CpG sites (cg09470983, cg11087101 and cg21494636) were significantly less methylated in BC tissues (Figure 7B). The heat map of the DNA methylation results for TfR1 in BC is shown in Figure 7C.

### Genomic mutations of TfR1 in BC

Genomic mutations are frequently and closely correlated with tumorigenesis. The results from the cBioPortal database indicated that there were approximately 2.4% genetic alterations in TfR1 in BC, including amplification, mutation and deep depletion (Supplementary Figure 3B, 3C). However, the KM plotter results showed that no statistically significant differences were found in the OS, DFS, PFS or DSS of BC patients with or without alterations in TfR1 (Supplementary Figure 3D).

### Identification of TfR1-interacting genes and proteins

**Figure 8:**
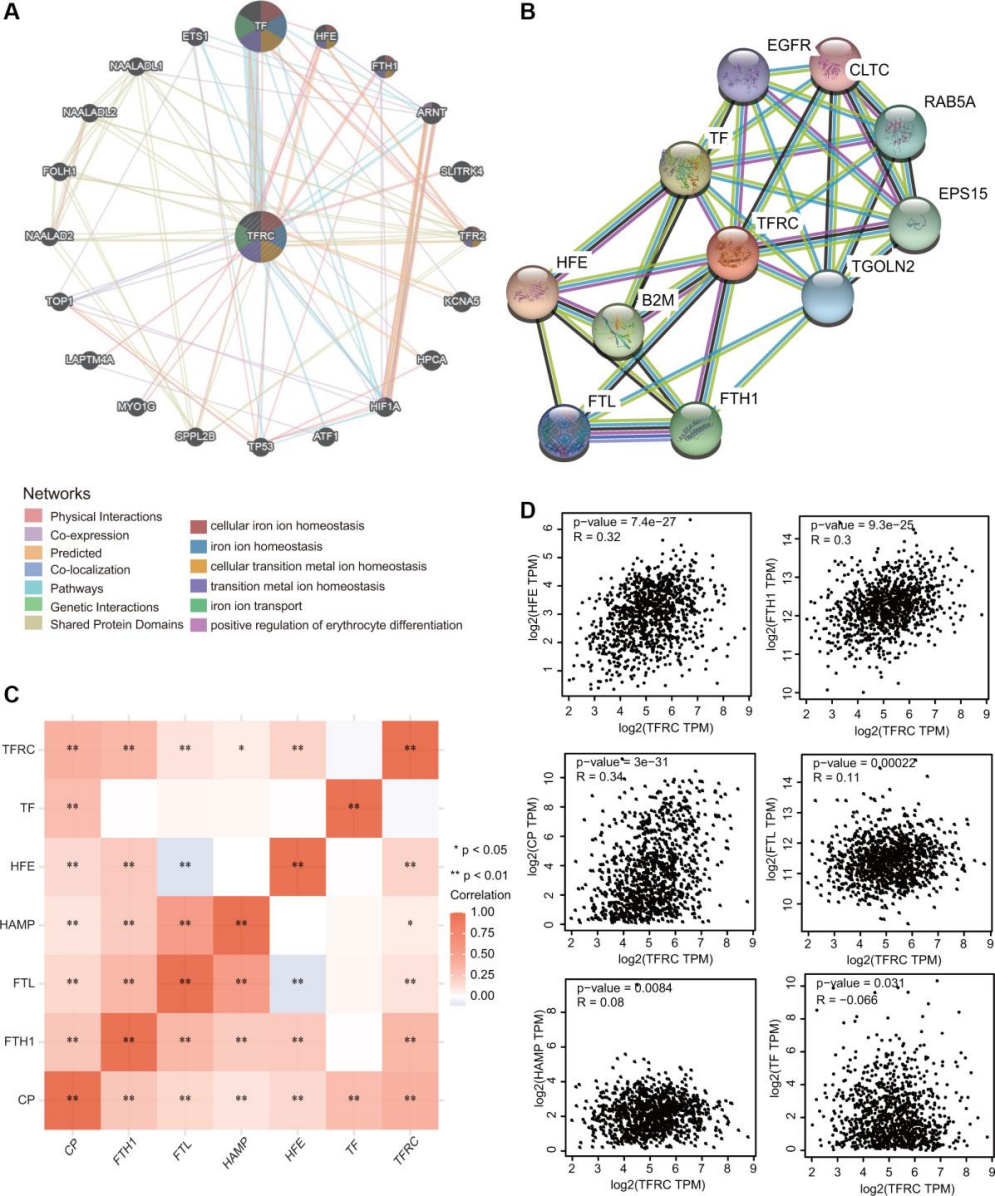
**Interaction network of TfR1 in BC.** (**A**, **B**) The gene-gene interaction and PPI network of TfR1 were constructed using GeneMANIA and STRING, respectively. (**C**) The heat map showing the correlations of TfR1 and various iron-related genes. (**D**) Scatterplots showing the correlations of TfR1 expression and HFE, FTH1, CP, FTL, HAMP and TF in BC through GEPIA database. ^*^< 0.05, ^**^< 0.01.

A gene-gene interaction network for TfR1 was first constructed to explore the mechanism of TfR1 in BC through the GeneMANIA database. Among the 20 genes associated with TfR1, the top five genes are TF, HFE, FTH1, ARNT and SLITRK (Figure 8A). Further functional analysis indicated that TfR1 and its-associated proteins were significantly correlated with cellular iron ion homeostasis, iron ion transport and positive regulation of erythrocyte differentiation (Figure 8A). To further investigate the function of TfR1, we constructed a PPI (protein-protein interaction) network containing 11 nodes and 35 edges through the STRING database (Figure 8B). The five nodes with the highest degree centrality were HFE, TF, B2M, FTH1 and EPS15 (Figure 8B). Interestingly, TfR1 was significantly and positively correlated with most iron metabolism genes, including CP, FTH1, FTL, HAMP and HFE (Figure 8C). The relationships between TfR1 and these genes were further evaluated through the GEPIA database (Figure 8D).

### Molecular mechanisms of TfR1 in BC

**Figure 9:**
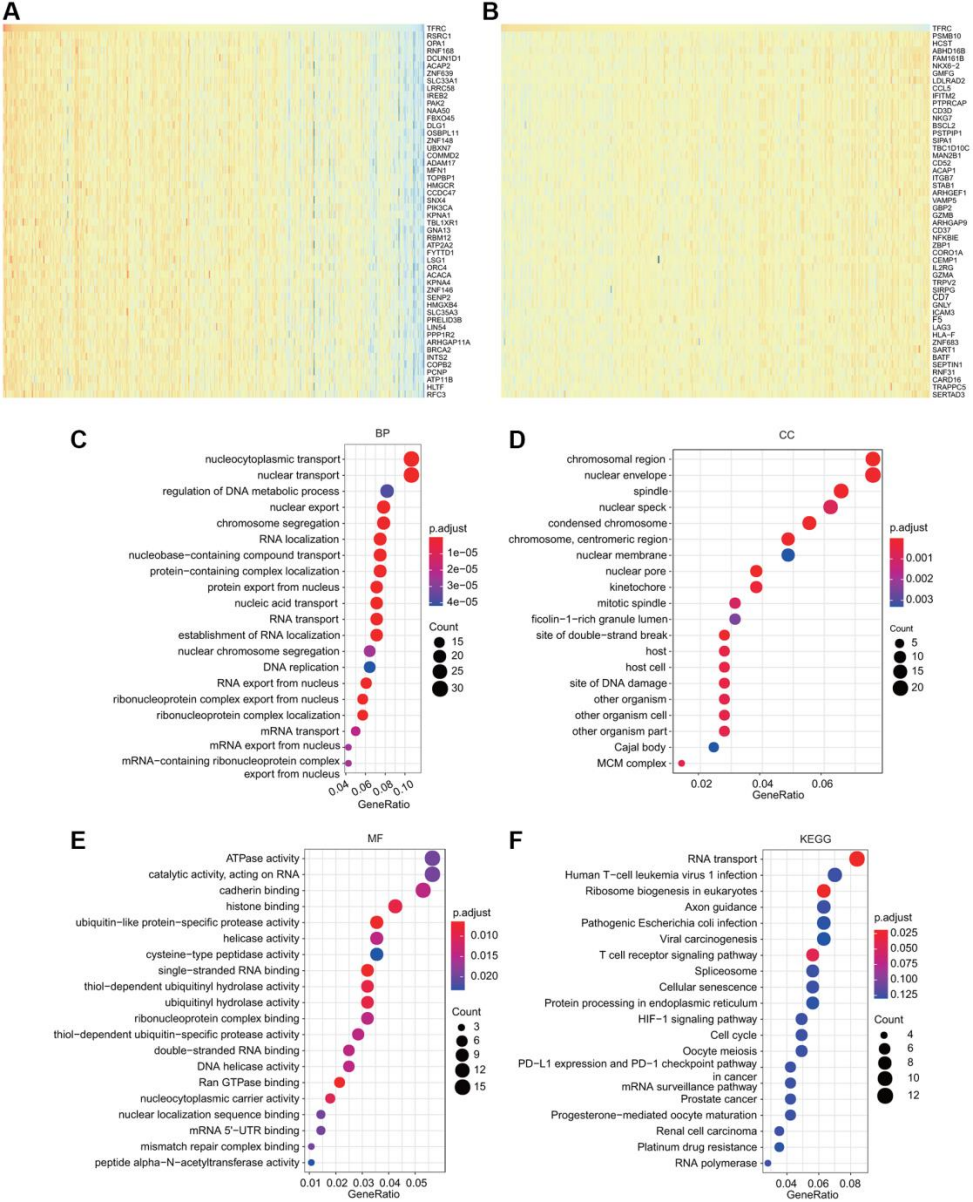
**GO and KEGG analyses for TfR1 in BC.** (**A**–**B**) Heat map showing the top genes that were coexpressed with TfR1 in BC. (**C**–**E**) Top twenty enriched signaling pathways in the BP, MF and CC in BC were shown. (**F**) Top twenty enriched signaling pathways based on the KEGG analysis were shown.

To deeply explore the molecular mechanisms and cellular functions of TfR1 in BC, a total of 300 TfR1-related genes obtained from the TCGA dataset were used to perform GO and KEGG analyses. The heat maps showed the top 50 genes that were positively or negatively coexpressed with TfR1 in BC (Figure 9A, 9B). The top five enriched biological process (BP) terms were nucleocytoplasmic transport, nuclear transport, regulation of DNA metabolic process, nuclear export, and chromosome segregation (Figure 9C). The top five enriched cellular component (CC) terms were nuclear envelope, spindle, chromosomal region, nuclear speck and nuclear membrane (Figure 9D). The top five enriched molecular function (MF) terms were ATPase activity, catalytic activity, cadherin binding, histone binding, and ubiquitin-like protein-specific protease activity (Figure 9E). Moreover, KEGG pathway analysis revealed that TFR1 was associated with pathways linked with oncogenesis, such as the HIF-1 signaling pathway, viral carcinogenesis, the cell cycle, prostate cancer, renal cell carcinoma and platinum drug resistance (Figure 9F). Additionally, the results of KEGG pathway also indicated that TfR1 was associated with the immune response, including the T cell receptor signaling pathway, human T-cell leukemia virus 1 infection, pathogenic *Escherichia coli* infection, PD-L1 expression and the PD-1 checkpoint pathway in cancer (Figure 9F). These findings suggest that TfR1 plays some roles in tumor development and the cancer-related immune response.

### TfR1-associated signaling pathways identified by gene set enrichment analysis (GSEA)

**Figure 10:**
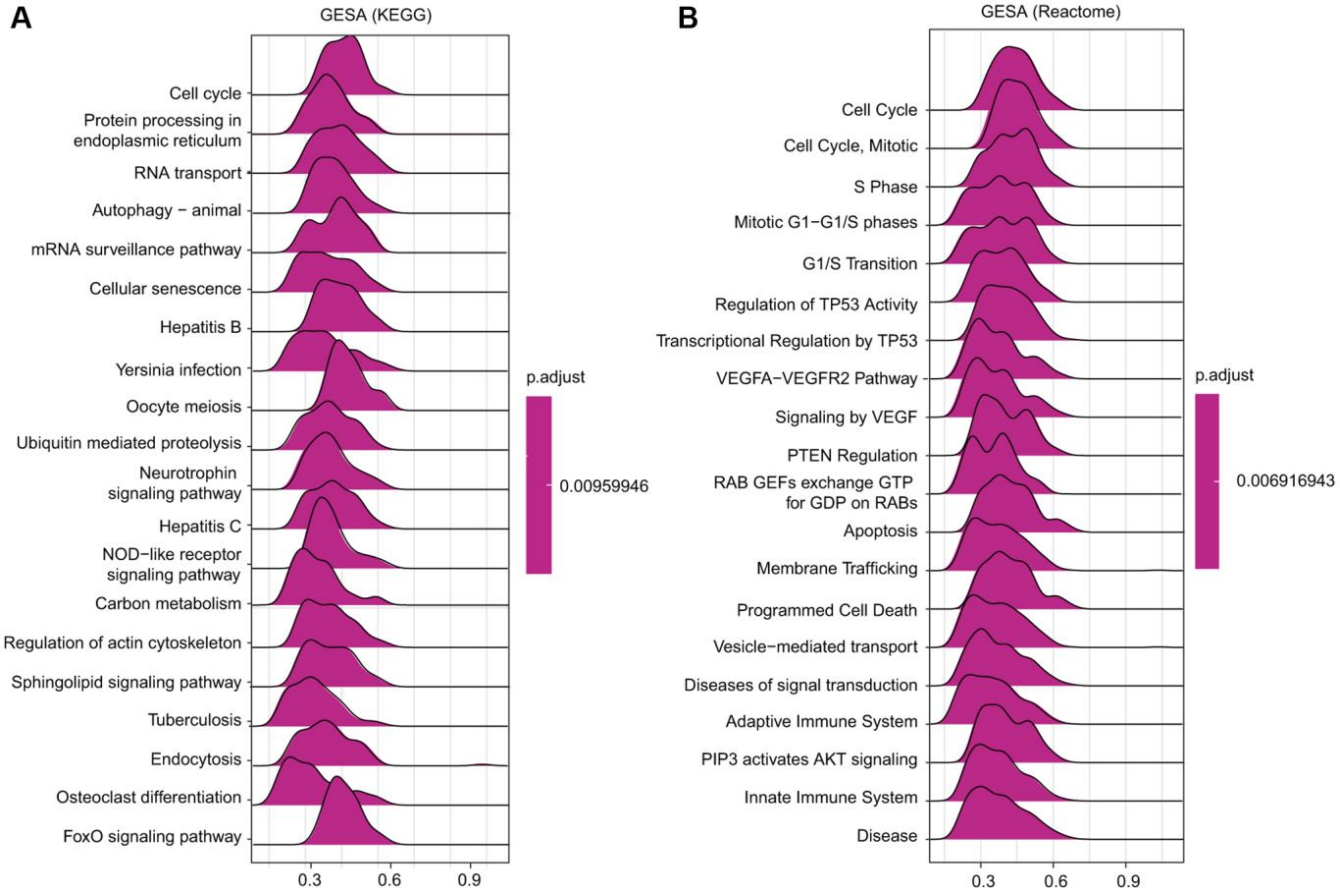
**Merged enrichment plots obtained by GSEA.** (**A**–**B**) Merged plots showing the enriched signaling pathways correlated with TfR1 expression based on KEGG analysis and Reactome analysis in BC.

Among the KEGG and Reactome terms of GSEA, many signaling pathways affected by TfR1 were enriched in tumorigenesis. In addition, several pathways were related to the immune response, including hepatitis B, Yersinia infection, hepatitis C and the NOD-like receptor signaling pathway, among the KEGG terms in BC (Figure 10A). Moreover, among the Reactome terms, antigen processing, class I MHC-mediated antigen processing and presentation, and adaptive immune system were significantly associated with TfR1 in BC (Figure 10B). These findings demonstrate that TfR1 may be closely related to the immune response in BC.

### Relationships between TfR1 expression and infiltration of different immune cells

**Figure 11:**
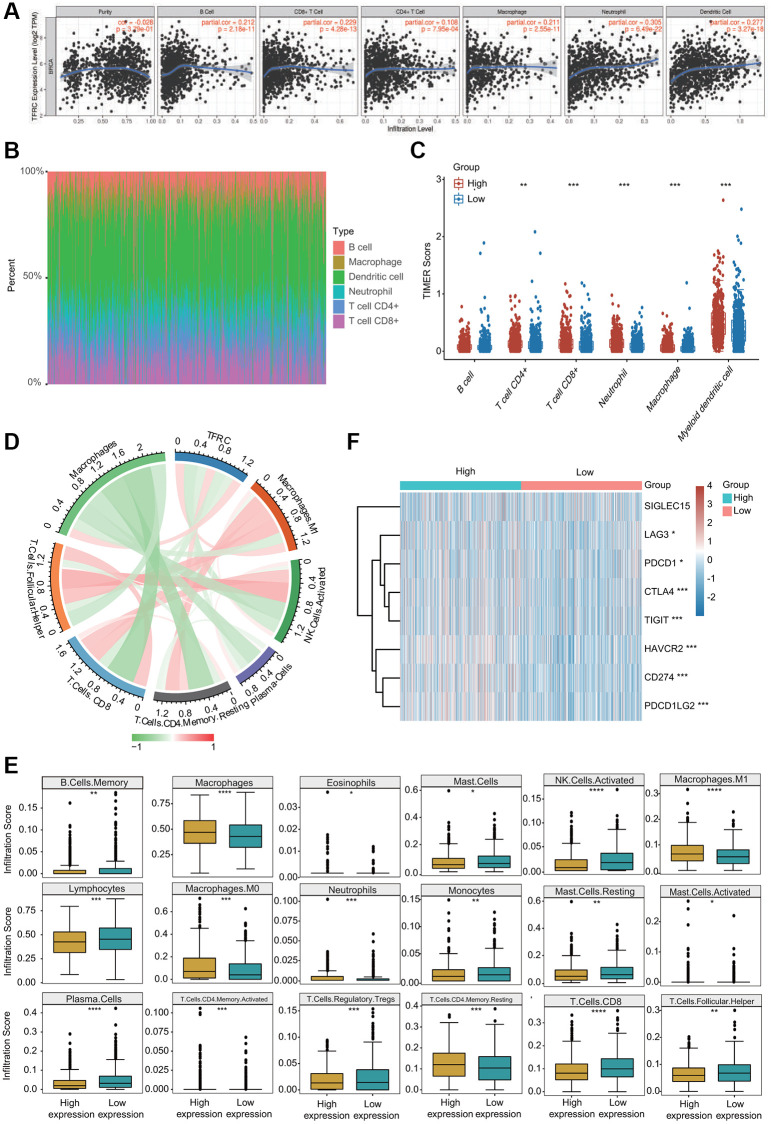
**Association between TfR1 expression and infiltration abundances of diverse immune cells in BC.** (**A**) TfR1 expression was significantly and positively correlated with the infiltration abundances of six types of immune cells in BC in the TIMER database. (**B**) Visualization of the infiltrating levels of multiple immune cells in the BC samples. (**C**) Comparison of the proportions of immune cells in the TfR1 high-expression and low-expression groups. (**D**) TfR1 expression was significantly associated with the infiltration abundances of diverse immune cells in BC using the CIBERSORT algorithm. (**E**) Correlations between TfR1 expression and the infiltration abundances of selected immune cells in BC through CIBERSORT algorithm. (**F**) The expression of various immune checkpoint genes between the TfR1 low-expression group and the high-expression group. ^*^p < 0.05, ^**^p < 0.01, ^***^p < 0.001.

Growing evidence has suggested that tumor infiltrating immune cells are potential predictors of cancer progression and survival in BC patients [4, 16]. We thus assessed the relationships between TfR1 expression and six major types of infiltrating immune cells. TfR1 exhibited strong positive correlations with the infiltration abundance of CD4+ T cells, CD8+ T cells, B cells, macrophages, neutrophils and dendritic cells in BC (Figure 11A). Based on TfR1 expression, BC patients were separated into high-expression and low- expression groups. The percentage abundance of tumor infiltrating immune cells in each sample with different colors and different types of immune cells using TIMER is shown (Figure 11B). Additionally, the infiltrating abundance of CD8+ T cells, CD4+ T cells, neutrophils, macrophages and dendritic cells was increased in the TfR1 high-expression group compared with the low-expression group (Figure 11C). The correlations of TfR1 expression with tumor-infiltrating immune cells in BC patients using the established analytical tool CIBERSORT were further analyzed. Importantly, TfR1 expression was positively associated with the infiltration of naïve B cells, memory CD4+ T cells, resting memory CD4+ T cells, macrophages, M0 macrophages, M1 macrophages and neutrophils but negatively associated with the infiltration of CD8+ T cells, naïve CD4+ T cells, memory B cells, lymphocytes, mast cells, resting mast cells, natural killer (NK) cells and plasma cells (Figure 11D and Supplementary Figure 4A, 4B). In addition, downregulated TfR1 expression was observed to be significantly associated with high infiltration scores of lymphocytes, memory B cells, mast cells, resting mast cells, monocytes, Treg cells, TfH cells, CD8+ T cells, activated NK cells and plasma cells but with low infiltration scores of eosinophils, macrophages, M0 macrophages, M1 macrophages, activated memory CD4+ T cells, activated mast cells, neutrophils and resting memory CD4+ T cells (Figure 11E). Taken together, these findings reveal that TfR1 expression is significantly correlated with tumor-infiltrating immune cells in BC patients.

### Correlations between TfR1 and various gene signatures of immune cells

The correlations of TfR1 expression and diverse marker sets of immune cell subsets in BC were assessed using the TIMER and GEPIA databases. TfR1 expression was remarkably associated with most gene markers of different immune cells, such as CD4+ T cells, CD8+ T cells, B cells, monocytes, tumor-associated macrophages (TAMs), neutrophils, M1 and M2 macrophages, dendritic cells and NK cells (Table 1). Similar relationships between TfR1 expression and diverse immune markers were obtained using the GEPIA database (Table 2).

**Table 1:** Correlations between TfR1 and different gene markers of immune cells in TIMER.

**Description**	**Gene markers**	**BC**			
		**None**		**Purity**	
		**Cor**	***P***	**Cor**	***P***
**Monocyte**	CSF1R	0.179	^***^	0.187	^***^
	CD86	0.3	^***^	0.325	^***^
**T cell (general)**	CD3D	0.044	0.146	0.046	0.147
	CD3E	0.064	^*^	0.072	^*^
	CD2	0.113	^***^	0.127	^***^
**CD8+ T cell**	CD8A	0.074	^*^	0.084	^**^
	CD8B	0.05	0.098	0.06	0.057
**B cell**	CD19	0.031	0.308	0.022	0.479
	CD79A	0.036	0.229	0.03	0.339
**M1**	PTGS2	0.136	^***^	0.149	^***^
	NOS2	0.048	0.113	0.048	0.13
	IRF5	0.078	^**^	0.079	^*^
**M2**	MS4A4A	0.228	^***^	0.25	^***^
	VSIG4	0.197	^***^	0.207	^***^
	CD163	0.341	^***^	0.363	^***^
**Neutrophils**	ITGAM	0.247	^***^	0.25	^***^
	CEACAM8	0.019	0.521	0.019	0.556
	CCR7	0.089	^**^	0.102	^**^
**TAM**	CCL2	0.167	^***^	0.178	^***^
	IL10	0.293	^***^	0.319	^***^
	CD68	0.31	^***^	0.336	^***^
**Dendritic cell**	HLA-DRA	0.202	^***^	0.223	^***^
	HLA-DQB1	0.075	^*^	0.075	^**^
	NRP1	0.194	^***^	0.2	^***^
	HLA-DPA1	0.134	^***^	0.147	^***^
	ITGAX	0.218	^***^	0.236	^***^
	HLA-DPB1	0.011	0.709	0.001	0.963
	CD1C	0.003	0.916	-0.018	0.568
**Natural killer cell**	KIR2DL4	0.136	^***^	0.142	^***^
	KIR2DL3	0.13	^***^	0.126	^***^
	KIR2DL1	0.079	^**^	0.088	^**^
	KIR3DL1	0.118	^***^	0.118	^***^
	KIR2DL4	0.136	^***^	0.142	^***^
	KIR2DS4	0.045	0.136	0.054	0.0893
	KIR3DL3	0.043	0.155	0.04	0.211

Abbreviation: TAM: tumor-associated macrophage. ^*^< 0.05, ^**^< 0.01, ^***^< 0.001.

**Table 2:** Correlations between TfR1 and different gene markers of immune cells in GEPIA.

**Description**	**Gene markers**	**BC**	
		**Purity**	
		***R***	***P***
**Monocyte**	CSF1R	0.21	^***^
	CD86	0.33	^***^
**T cell (general)**	CD3D	0.024	0.43
	CD3E	0.047	0.06
	CD2	0.12	^***^
**CD8+ T cell**	CD8A	0.012	0.076
	CD8B	0.05	0.1
**B cell**	CD19	0.017	0.58
	CD79A	0.03	0.32
**M1**	PTGS2	0.17	^***^
	NOS2	0.099	^***^
	IRF5	0.12	^***^
**M2**	MS4A4A	0.25	^***^
	VSIG4	0.2	^***^
	CD163	0.24	^***^
**Neutrophils**	CEACAM8	0.023	0.45
	ITGAM	0.28	^***^
	CCR7	0.1	^***^
**TAM**	CCL2	0.17	^***^
	IL10	0.32	^***^
**Dendritic cell**	HLA-DRA	0.21	^***^
	HLA-DQB1	0.029	0.34
	NRP1	0.25	^***^
	HLA-DPA1	0.17	^***^
	ITGAX	0.23	^***^
	HLA-DPB1	0.047	0.12
	CD1C	–0.0074	0.81
	KIR2DL4	0.14	^***^
**Natural killer cell**	KIR2DL3	0.13	^***^
	KIR2DL1	0.11	^***^
	KIR3DL1	0.092	^**^
	KIR2DS4	0.027	0.38
	KIR3DL2	0.14	^***^
	KIR3DL3	0.073	^**^

Abbreviation: TAM: tumor-associated macrophage. ^*^< 0.05, ^**^< 0.01, ^***^< 0.001.

More importantly, TfR1 expression was strongly associated with 34 of 36 T cell markers of multiple types of functional T cells (Table 3). In addition, TfR1 expression was remarkably associated with 33 of 36 T cell markers in BC after adjusting for tumor purity (Table 3).

**Table 3:** Correlations between TfR1 and various gene markers of diverse types of T cells in TIMER.

**Description**	**Gene markers**	**BC**			
		**None**		**Purity**	
		**Cor**	***P***	**Cor**	***P***
**Th1**	TBX21	0.077	^**^	0.083	^**^
	STAT4	0.13	^***^	0.142	^***^
	STAT1	0.366	^***^	0.376	^***^
	IFNG	0.151	^***^	0.169	^***^
	TNF	0.23	^***^	0.223	^***^
	GATA3	0.173	^***^	–0.19	^***^
**Th2**	STAT6	0.053	0.076	–0.057	0.07
	STAT5A	0.046	0.131	0.035	0.268
	IL13	0.068	^**^	0.079	^*^
**Tfh**	BCL6	0.158	^***^	0.157	^***^
	IL21	0.165	^***^	0.169	^***^
**Th17**	STAT3	0.357	^***^	0.357	^***^
	IL17A	0.068	^**^	0.058	0.065
**Treg**	FOXP3	0.222	^***^	0.244	^***^
	CCR8	0.347	^***^	0.366	^***^
	STAT5B	0.088	^**^	0.085	^**^
	TGFB1	0.026	0.392	–0.037	0.245
	CX3CR1	–0.035	0.242	–0.062	0.051
**Naïve T-cell**	SELL	0.12	^***^	0.117	^***^
	TCF7	0.129	^***^	0.129	^***^
	LEF1	–0.017	0.582	–0.027	0.39
**Effector T-cell**	FGFBP2	–0.031	0.309	–0.042	0.184
	FCGR3A	0.32	^***^	0.339	^***^
**Effective Treg T-cell**	CCR7	0.089	^**^	0.102	^**^
	FOXP3	0.222	^***^	0.244	^***^
	CTLA4	0.191	^***^	0.211	^***^
	CCR8	0.347	^***^	0.366	^***^
**Resident memory T-cell**	TNFRSF9	0.381	^***^	0.408	^***^
	IFNG	0.151	^***^	0.169	^***^
	CD69	0.106	^***^	0.123	^***^
	ITGAE	0.143	^***^	0.142	^***^
	CXCR6	0.183	^***^	0.203	^***^
**Exhausted T-cell**	MYADM	0.123	^***^	0.138	^***^
	HAVCR2	0.303	^***^	0.324	^***^
	TIGIT	0.205	^***^	0.23	^***^
	LAG3	0.087	^**^	0.095	^**^
	PDCD1	0.062	^*^	0.066	^*^
	CXCL13	0.155	^***^	0.161	^***^
**Resting Treg T-cell**	LAYN	0.034	0.255	0.019	0.559
	FOXP3	0.222	^***^	0.244	^***^
	IL2RA	0.323	^***^	0.353	^***^
**Effective memory T-cell**	PDCD1	0.062	^*^	0.066	^*^
	DUSP4	–0.001	0.976	–0.011	0.739
	GZMK	0.027	0.367	0.029	0.364
	GZMA	0.042	0.162	0.045	0.154
**Th1-like**	CXCL13	0.155	^***^	0.161	^***^
	HAVCR2	0.303	^***^	0.324	^***^
	IFNG	0.151	^***^	0.169	^***^
	CXCR3	0.049	^***^	0.05	0.115
	BHLHE40	0.039	0.198	0.037	0.246
	CD4	0.211	^***^	0.233	^***^
	CCR7	0.089	^**^	0.102	^**^
**General memory T-cell**	SELL	0.12	^***^	0.117	^***^
	IL7R	0.314	^***^	0.358	^***^

^*^< 0.05, ^**^< 0.01, ^***^< 0.001.

The relationships between TfR1 expression and well-known immune checkpoints were evaluated to estimate the immunotherapy responses associated with TfR1 expression. Significantly higher expression of LAG3, CTLA-4, PDCD-1 (PD-L1), CD274 (PD-1), TIGIT, HAVCR2 and PDCD1LG2 was observed in the TfR1 high-expression group than in the TfR1 low-expression group (Figure 11F). The expression of TfR1 was also associated with the expression of CTLA-4, PD-L1 and PD-1 in BC according to data from the GEPIA and TIMER databases (Supplementary Figure 5A). Interestingly, we also observed that the expression of TfR1 was remarkably upregulated in BC patients with different treatments, such as chemotherapy, hormone therapy and immunotherapy (Supplementary Figure 5B).

### Prognostic significance of TfR1 based on immune cell infiltration in BC patients

**Figure 12:**
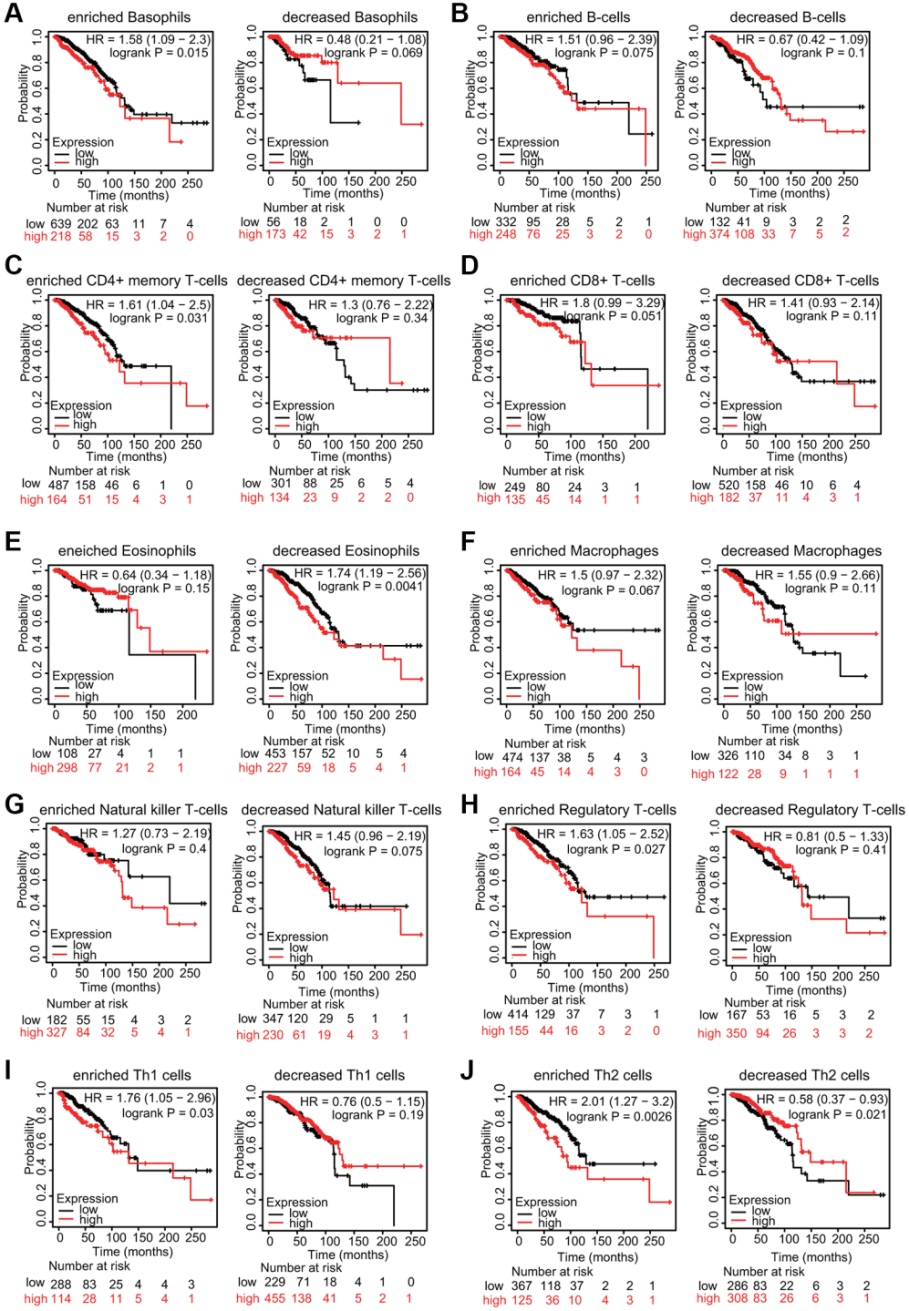
**KM survival curves based on different expression levels of TfR1 in various subgroups of BC patients.** (**A**–**J**) The relationship between TfR1 expression and the OS rate in diverse immune cell subgroups of BC patients was explored.

Because increased TfR1 expression was linked to poor prognosis and immune infiltration plays a role in patient outcomes, the associations of TfR1 expression and the prognosis of BC patients in different immune cell subgroups were examined. KM plotter results indicated that BC patients with high TfR1 expression and increased infiltration of basophils, memory CD4+ T cells, regulatory T cells, Th1 cells and Th2 cells exhibited unfavorable OS (Figure 12A, 12C, 12H–12J). In addition, BC patients with high TfR1 expression and decreased infiltration of eosinophils had poor OS (Figure 12E). Moreover, there was no significant relationship between TfR1 expression and the prognosis of BC patients in cohorts with either increased or decreased infiltration of B cells, CD8+ T cells, macrophages, and NK T cells (Figure 12B, 12D, 12F, 12G). These findings reveal that upregulated TfR1 expression can influence the prognosis of BC patients partially through the infiltration of immune cells.

## DISCUSSION

BC is the most common cancer type and the first leading cause of cancer-associated deaths in females worldwide [1–3]. Increasing evidence has demonstrated that alterations in iron metabolism-associated proteins play vital roles in BC [17–19]. Among various iron-related proteins, TfR1 expression was reported to be dysregulated in some types of human cancer, including BC. Habashy et al. reported that elevated TfR1 expression was related to cancer cell proliferation, poor NPI score, and poor survival of breast cancer patients [20]. Moreover, TfR1 was associated with HER2, P53, EGFR, ER and AR in BC. TfR1 could act as an independent prognostic factor in ER+/luminal-like BC patients based on multivariate analysis [20]. Miller et al. found that an iron anti-import phenotype with concomitant low TfR1 and high HFE was significantly associated with favorable prognosis of BC patients [21]. Jiang et al. demonstrated that TfR1 expression was much higher in breast cancer cells (MCF-7 cells) than in normal mammary epithelial cells (MCF-12A cells) [22]. Knocking down TfR1 not only reduced cellular iron levels but also suppressed the proliferation of breast cancer cells *in vitro* and tumor growth and lung metastases *in vivo* [22]. Wang et al. found that although the iron regulatory proteins IRP1 and IRP2 were upregulated in BC, only IRP2 expression was correlated with FTH1 and TfR1 expression [23]. Silencing IRP2 greatly retarded breast tumor growth *in vivo* by decreasing TfR1 expression and increasing FTH1 expression to reduce the labile iron pool [23]. The TfR1 protein level in the preclinical plasma of BC patients was different from that in matching controls, and TfR1 may be a novel plasma protein biomarker for ER-positive/PR-positive invasive ductal carcinoma [24]. Marques et al. found that TfR1 expression was increased in epithelial cells, macrophages and lymphocytes from breast carcinoma samples compared with that in normal controls [10]. In addition, TfR1 expression was higher in infiltrating lymphocytes and macrophages in invasive ductal carcinoma samples than in ductal carcinoma samples *in situ* [10]. Consistent with these previous studies, we also found that TfR1 mRNA and protein expression levels were remarkably upregulated in BC tissues (Figures 1 and 2). KM analyses and Cox regression analyses based on clinicopathological characteristics suggested that TfR1 expression was an independent prognostic factor of the survival of BC patients (Figures 5 and 6). Additionally, a nomogram was constructed to further support that TfR1 may be an indicator for the diagnosis of BC (Figure 6C).

TfR1 expression is mediated at both the transcriptional and posttranscriptional levels. The basal expression of TfR1 is regulated by Sp1, which can bind to the GC-rich region in the TfR1 promoter [25]. In erythroid cells, EST-1 is also involved in TfR1 regulation [26]. In response to iron deficiency or hypoxic conditions, the protein expression of hypoxia-inducible factors, including HIF-1α and HIF-2α, is significantly elevated, and these proteins are recruited to the promoter of TfR1, thereby increasing TfR1 transcription [27, 28]. Interestingly, TfR1 expression was influenced by the circadian clock in mouse colon cancer cells [29]. The clock-controlled oncogene c-MYC rhythmically promoted the activation of TfR1 transcription [29, 30]. At the posttranscriptional level, a sophisticated mechanism, called the IRP1/2-IRE (iron responsive element) system, regulates iron homeostasis by modulating TfR1 expression [5, 31]. Under iron depletion conditions, IRP1/2 binds to IREs to stabilize the mRNA of TfR1 and increase protein expression. Under iron overload conditions, IRP1 changes to an aconitase through conformational alterations, and IRP2 will be degraded in a ubiquitination-dependent manner. As a result, the interaction between IRP1/2 and IREs disappears, leading to the destabilization and degradation of TfR1 mRNA [5, 31]. Moreover, microRNA-107 (miR-107) prevented the proliferation and invasion of colorectal cancer cells by negatively mediating TfR1 expression in colorectal cancer [32]. TfR1 expression is significantly upregulated in liver cancers and related to unfavorable survival [33]. miR-152 could effectively inhibit TfR1 expression, indicating that miR-152-targeted TfR1 expression may become one of novel anticancer therapeutic approaches for liver cancer [33]. Interestingly, E2-induced DNA methylation downregulated the expression of TfR1 in both Hep-G2 and Huh7 cells, and silencing TfR1 caused cell cycle arrest, ROS overproduction, decreased proliferation and increased apoptosis in live cancer cells [34]. Additionally, the activation of sphingosine kinase 1 (SK1) increased TFR1 expression, leading to the enhanced uptake of transferrin into cells [35]. Notably, inhibition of TfR1 with a neutralizing antibody significantly prevented SK1-regulated cell growth, cell survival and neoplastic transformation, indicating that TfR1 is a downstream effector in SK1-mediated oncogenesis [35]. TfR1 antisense oligonucleotide blocked tumor growth and lung metastasis in the 4T1 mammary adenocarcinoma mouse model [22]. Transferrin promotes the formation of histone 2AX phosphorylated at Ser139 (γH2AX), a classical DNA damage marker [36]. Silencing of TfR1, but not TfR2, inhibited transferrin uptake, ROS generation and the consequent formation of γH2AX [36]. Thus, the transferrin-TfR1 axis may facilitate carcinogenesis by triggering DNA damage and genome instability. Furthermore, epidermal growth factor receptor (EGFR) could exert its oncogenic ability by binding to and mediating TfR1 cellular distribution [37]. The inactivation of EGFR decreases the expression of cell surface TfR1, which results in reduced import of iron and subsequent cell cycle arrest and proliferation inhibition [37].

More importantly, in the present study, GO, KEGG and GSEA results indicate that TfR1 is associated with multiple signaling pathways, including the immune response (Figuress 8 and 9A). In addition, we analyzed the correlations between TfR1 expression and the infiltration abundances of diverse immune cells in BC and observed that TfR1 was positively and significantly correlated with the infiltration scores of CD4+ T cells, CD8+ T cells, B cells, neutrophils, macrophages, and dendritic cells in BC (Figure 10A). Previously, TfR1 was shown to be involved in the formation of immunological synapses in T cells in response to TCR engagement and in T-cell receptor function by inducing tyrosine phosphorylation [38]. RNA-sequencing results from TfR1-overexpressing and normal control HeLa cells revealed that TfR1 regulated the expression of target genes associated with ion transport and immunity [39]. In addition, TfR1 was shown to interact with the IKK complex and was associated with IKK-NF-κB signaling, which is the major regulator of the immune response [40]. Compared with healthy subjects, idiopathic pulmonary fibrosis patients exhibit an increase in the proportion of airway macrophages lacking TfR1, which is characterized by impaired macrophage maturity, defective phagocytosis and increased profibrotic gene expression [41]. More importantly, airway macrophages lacking TfR1 were shown to be independently correlated with a worse survival rate [41]. Recently, both patients and a mouse model with a homozygous mutation in *TFRC* exhibited combined immunodeficiency, which is characterized by impaired proliferation of T and B cells and defective class switching, which is essential for antibody production [42, 43]. The homozygous p.Tyr20His substitution in TfR1 hindered TfR1 internalization and receptor endocytosis, leading to elevated TfR1 expression [42]. All these findings and our results reveal the important roles of TfR1 in modulating the immune response and immune cell infiltration in cancer (Figure 11). Interestingly, our results also suggest that TfR1 influences prognosis partially through immune infiltration in BC patients (Figure 12). Overall, these results provide an interrelationship and an underlying mechanism between TfR1 and immune infiltration in BC.

TfR1 has been explored as a potential therapeutic target due to its cell surface accessibility, constitutive endocytosis into cells, requirement for cell growth, and overexpression by cancer cells [13]. Among the developed therapeutic treatments targeting TfR1, antibodies stand out because of their exquisite specificity and high affinity [14, 15, 44]. TfR1 monoclonal antibodies, including 42/6, A24, JST-TFR09, and ch128.1Av (anti-hTfR1 IgG3-Av), facilitate the accumulation of nonselective therapeutic agents in cancer cells, elevate the intracellular drug concentration, improve anticancer activity, and enhance therapeutic efficacy [14, 15]. Among these antibodies, the TfR1 antibody 42/6 was proven to have the most potent cytotoxic effects against human tumors [45]. Additionally, chemotherapeutic agents may also affect TfR1 expression. Gefitinib and erlotinib are first-generation EGFR (epidermal growth factor receptor) tyrosine kinase inhibitors (TKIs) in the treatment of NSCLC patients with EGFR mutations. The protein expression of TfR1 was gradually decreased following gefitinib and erlotinib treatment for 48 and 72 h in human lung adenocarcinoma PC-9 cells [37]. Cisplatin, a platinum-based chemotherapy drug, is commonly used for the treatment of various types of cancer, including ovarian, bladder, lung, cervical, head and neck, and testicular cancer. A recent study revealed that cisplatin treatment disturbed iron metabolism to induce cytotoxicity [46]. Mechanistically, cisplatin inhibited the binding of IRP2 to the iron-responsive elements of TfR1 by binding IRP2 at Cys512 and Cys516. As a result, TfR1 transcription was significantly prevented, and iron depletion occurred. The combination of cisplatin and desferrioxamine, a clinical iron-chelating agent, further increased cytotoxicity by enhancing iron deficiency [46]. Moreover, TfR1 was highly expressed in retinoblastoma cells compared to normal retinal cells [47]. Artesunate exhibited cytotoxic effects, and its internalization in retinoblastoma cells was dependent on the expression of TfR1 at the membrane [47]. Increasing evidence suggests that dihydroartemisinin (DHA), a clinically used antimalarial agent, exhibits anticancer activity in numerous cancer cells [48, 49]. DHA treatment resulted in iron deficiency by decreasing the expression of cell-surface TfR1 through an endocytic pathway [48, 49]. Sulfasalazine, an anti-inflammatory drug, is extensively used in chronic, long-term therapy of inflammatory bowel disease (ulcerative colitis) and rheumatoid arthritis. Recently, sulfasalazine was reported to trigger ferroptosis in pancreatic and breast cancer cells [50]. Interestingly, TfR1 expression was upregulated in a dose-dependent manner during sulfasalazine treatment in breast cancer cells, indicating that activation of TfR1 may participate in sulfasalazine-induced ferroptosis [50]. Here, our analyses also found that the expression of TfR1 was remarkably increased in BC patients treated with chemotherapy, hormone therapy and immunotherapy (Supplementary Figure 5B). Advances in all these fronts reveal the enormous potential for the development of cancer therapies by targeting TfR1.

Taken together, our results suggest that high TfR1 expression is significantly correlated with the cancer progression and worse prognosis of BC and provide an interrelationship and an underlying mechanism between TfR1 and immune infiltration in BC.

## METHODS

### Oncomine analysis

The Oncomine database (http://www.oncomine.org) was used to examine the mRNA expression of TfR1 in BC. The search was performed according to the following criteria: (1) analysis type: cancer versus normal tissues; (2) data type: mRNA; and (3) thresholds: fold change > 1.5 and *P* value < 0.05.

### GO, KEGG and GSEA

GO, KEGG and GSEA were used to explore the biological and molecular functions of TfR1 in BC. GO analysis was applied to investigate the BP, CC and MF associated with TfR1. All three analyses were carried out using the R package Cluster Profiler.

### GEPIA database

In the present study, we examined the correlations between TfR1 and immune-related genes in the GEPIA database (http://gepia.cancer-pku.cn/). Values of *P* < 0.05 were applied.

### Kaplan-Meier (KM) plotter

To explore the potential prognostic significance of TfR1 in BC, the associations between TfR1 expression and survival analysis, including OS, PPS, RFS and DMFS of BC patients were performed by KM plotter (http://kmplot.com). The JetSet best probe set (208691_at) for TfR1 was selected. In addition, we also analyzed TfR1 prognostic values associated with clinicopathological parameters using KM plotter.

### bc-GenExMiner (BC Gene-Expression miner) v4.5

In the present study, the associations between TfR1 expression and the clinicopathological characteristics of BC were investigated through bc-GenExMiner v4.5.

### TIMER database analysis

The correlations between TfR1 expression and the infiltration of major immune cells in BC were assessed using the TIMER database (https://cistrome.shinyapps.io/timer/). Moreover, the relationships between TfR1 expression and different gene markers of various immune cells were also evaluated through this database.

### CIBERSORT algorithm

The relationships between TfR1 expression and the proportions of multiple tumor-infiltrating immune cells in BC were evaluated using the CIBERSORT algorithm. *P*-value < 0.05 was chosen as the threshold to select immune cells that were influenced by TfR1 expression.

### HPA (Human Protein Atlas) database

The HPA (http://www.proteinatlas.org) database was utilized for immunohistochemistry (IHC) validation of TfR1.

### UALCAN database

The mRNA expression and DNA methylation of TfR1 in BC tissues and in corresponding normal breast tissues were explored through the UALCAN database (http://ualcan.path.uab.edu/). Moreover, the expression profiles and DNA methylation of TfR1 according to clinicopathologic parameters, such as tumor grade, cancer stage and age, were also investigated through the UALCAN database.

### SurvivalMeth

The heatmap of CpG methylation levels of TfR1 in BC was also obtained from the SurvivalMeth database (http://bio-bigdata.hrbmu.edu.cn/survivalmeth/).

### GeneMANIA and STRING databases

GeneMANIA (http://www.genemania.org) and STRING (https://string-db.org/) were used to construct gene-gene and protein-protein interaction networks for TfR1 as previously described [4, 18, 19].

### cBioPortal database

The genomic profiles of TfR1 in BC, such as the genetic alterations and effect on survival curves, were examined using cBioPortal’s online database.

### Univariate and multivariate Cox regression analyses and construction of a nomogram

Cox regression analyses (univariate and multivariate analysis) were utilized to investigate the prognostic potential of TfR1 in BC. A forest plot was constructed to show the hazard ratio (HR), 95% CI and *p*-value using the R package “forest plot”. Additionally, a nomogram was constructed using the R package “rms”.

### Statistical analysis

TfR1 expression in cancer tissues and normal tissues was compared using Student’s *t*-test, Wilcox. test or Kruskal-Wallis test. The relationships between TfR1 and the immune infiltration level in CIBERSORT and heat maps showing genes coexpressed with TfR1 in TCGA were analyzed using Pearson’s correlation. Moreover, Pearson’s correlation and Spearman’s correlation were used to assess the correlations between different genes. *P* < 0.05 was considered statistically significant.

## Supplementary Materials

Supplementary Figures
