# Supplementary material for: Integrated analysis identifies TfR1 as a prognostic biomarker which correlates with immune infiltration in breast cancer
Source: Aging (Albany NY). 2021 Sep 13;13(17):21671–99. doi: 10.18632/aging.203512 (PMC8457555; doi:10.18632/aging.203512)
Supplement: Supplementary Figures [file aging-13-203512-s001.pdf]

SUPPLEMENTARY FIGURES

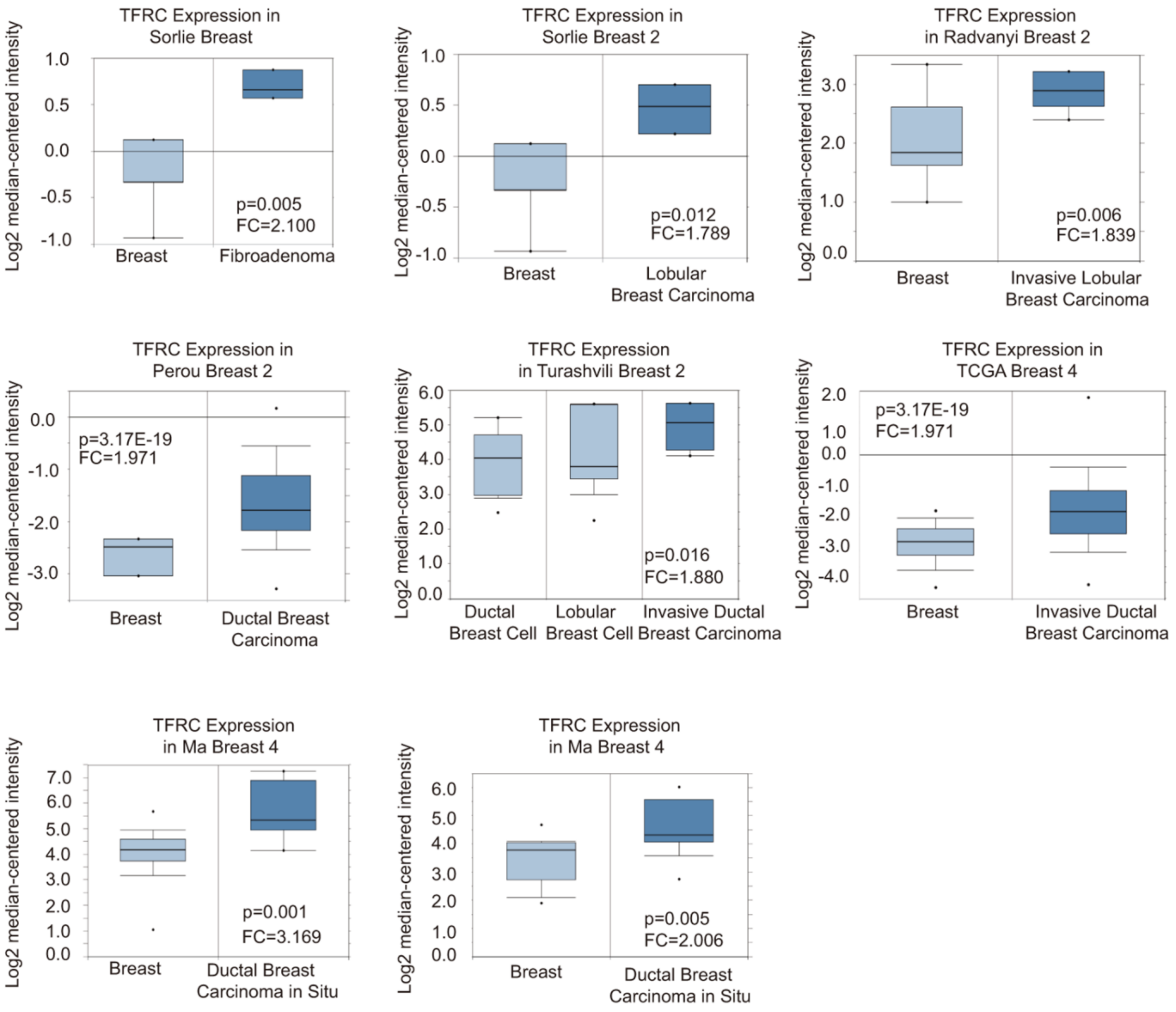

Supplementary Figure 1. Box plots showing TfR1 expression in BC patients with different types and normal individuals through the Oncomine database.

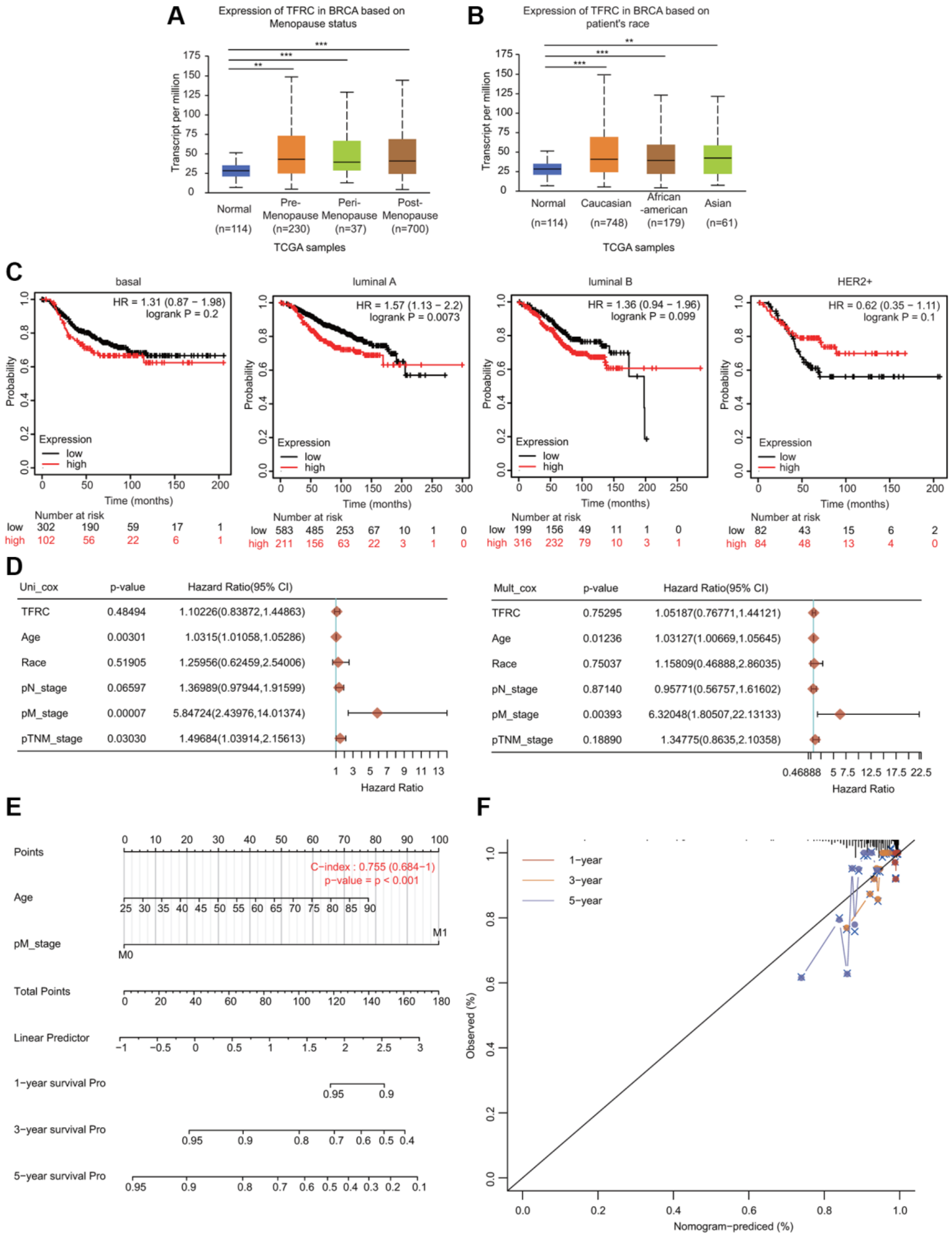

Supplementary Figure 2. Relationships between the expression of TfR1 and clinicopathological characteristics of BC patients. TfR1 expression was investigated in (**A**) different menopausal patients (normal individuals, *n* = 114; premenopausal, *n* = 230; perimenopausal, *n* = 37; and postmenopausal, *n* = 700) and (**B**) patients of different races (normal individuals, *n* = 114; Asian, *n* = 61; African-American, *n* = 179; and Caucasian, *n* = 748). (**C**) The OS of different subtypes of BC (basal, luminal A, luminal B and HER2+) was obtained from the KM plotter database. (**D**) Univariate and multivariate Cox regression analyses determined TfR1 as an independent prognostic factor in the luminal A subtype of BC. (**E**) A prognostic nomogram integrating TfR1 expression and clinicopathologic variables was constructed to estimate OS in luminal A subtype of BC. (**F**) Calibration plots to predict the OS of luminal A subtype patients at 1, 3, and 5 years. $^{*}< 0.05$, $^{**}< 0.01$, $^{***}< 0.001$.

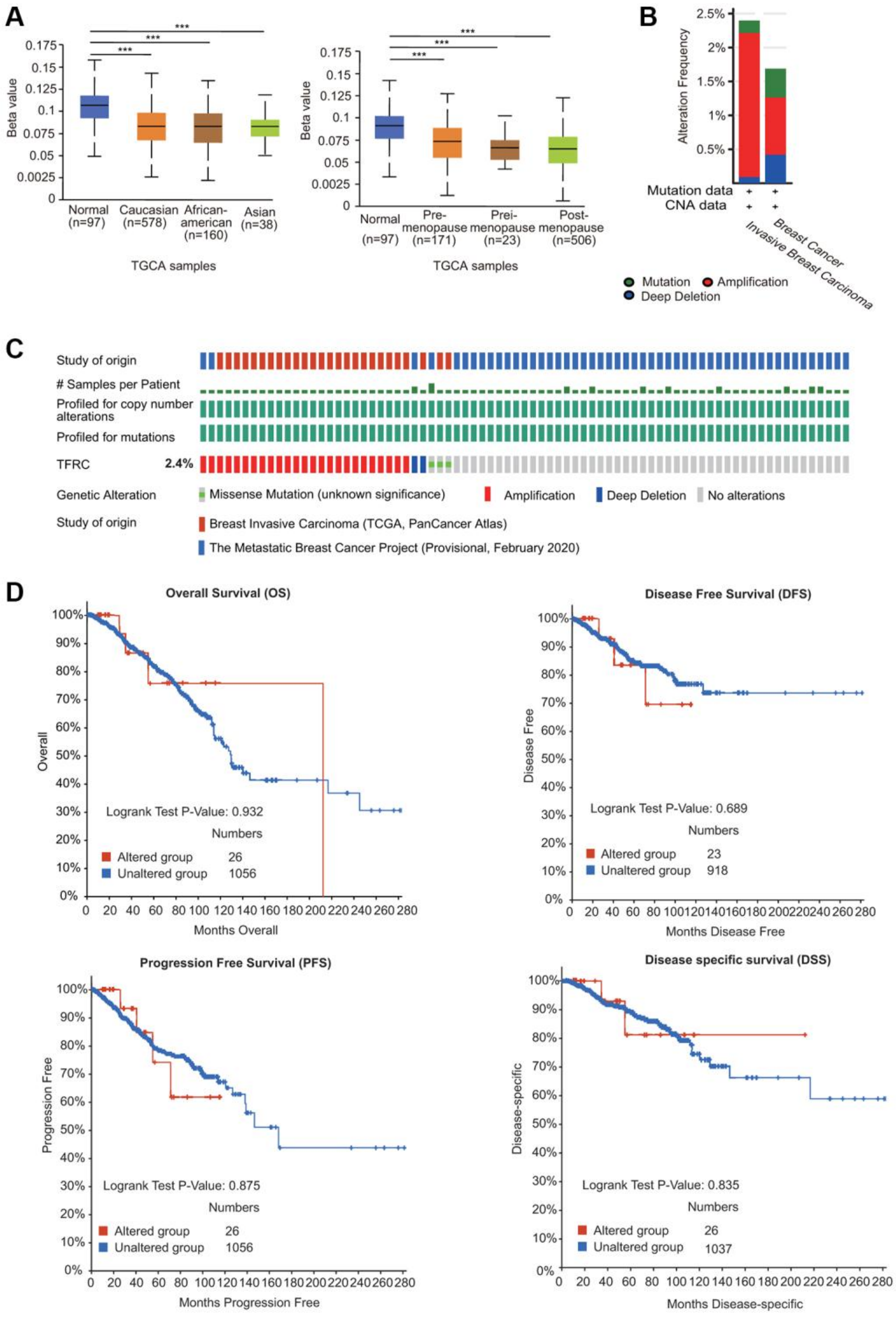

Supplementary Figure 3. (**A**) Association of DNA methylation of TfR1 with clinicopathological parameters of BC patients. The DNA methylation of TfR1 was investigated in patients of different races (normal individuals, *n* = 97; Asian, *n* = 38; African-American, *n* = 160; and Caucasian, *n* = 578) and different menopausal patients (normal individuals, *n* = 97; premenopausal, *n* = 171; perimenopausal, *n* = 23; and postmenopausal, *n* = 506). (**B**) Alteration frequency of TfR1 in BC from the cBioPortal database. (**C**) Summary of changes in a query of TfR1. (**D**) KM plots of OS, DFS, PFS and DSS in BC patients with or without gene alterations in TfR1. *< 0.05, **< 0.01, ***< 0.001.

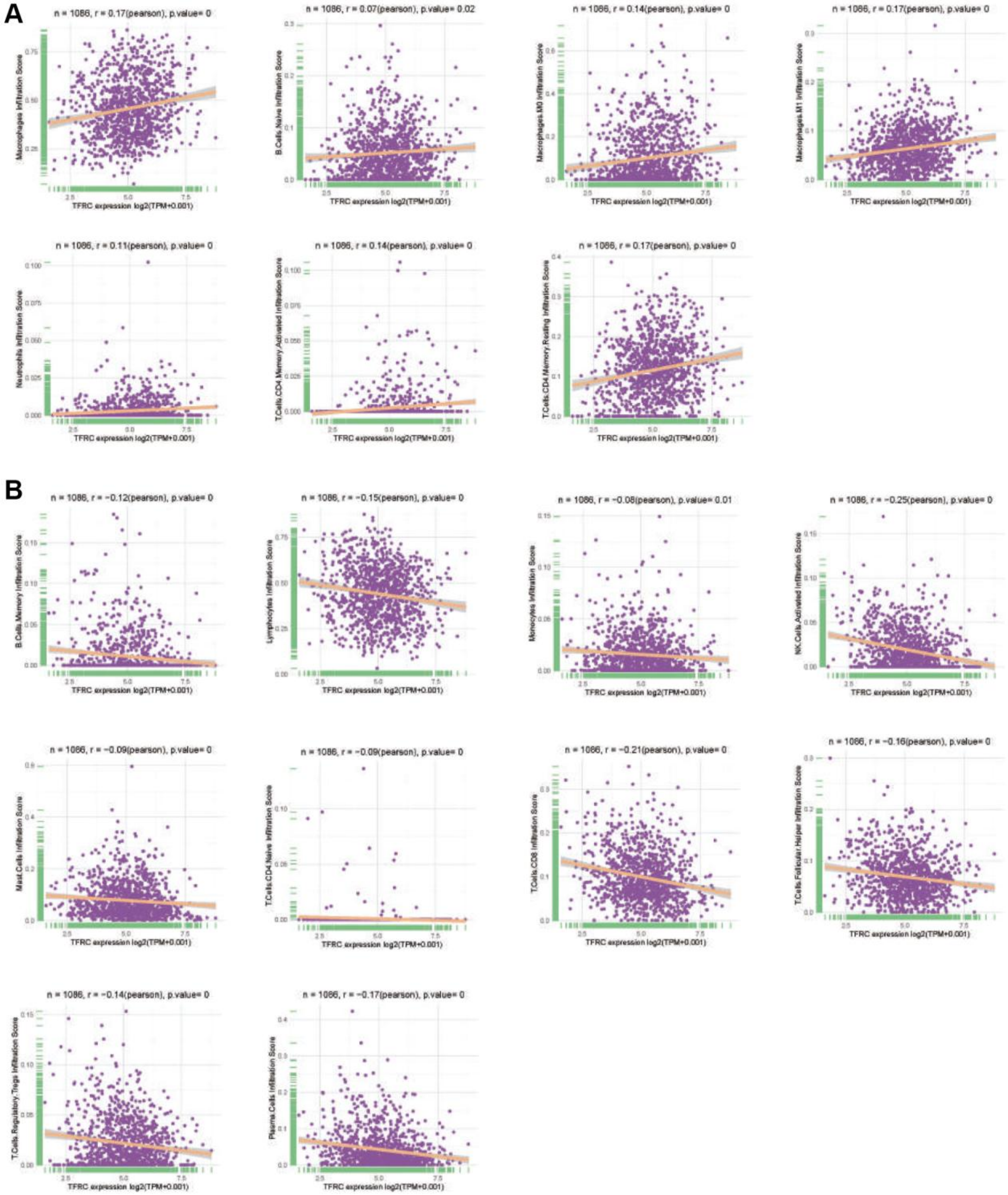

Supplementary Figure 4. (**A**, **B**) Scatterplots of positive or negative correlations between the expression of TfR1 and infiltration scores of different immune cells in BC.

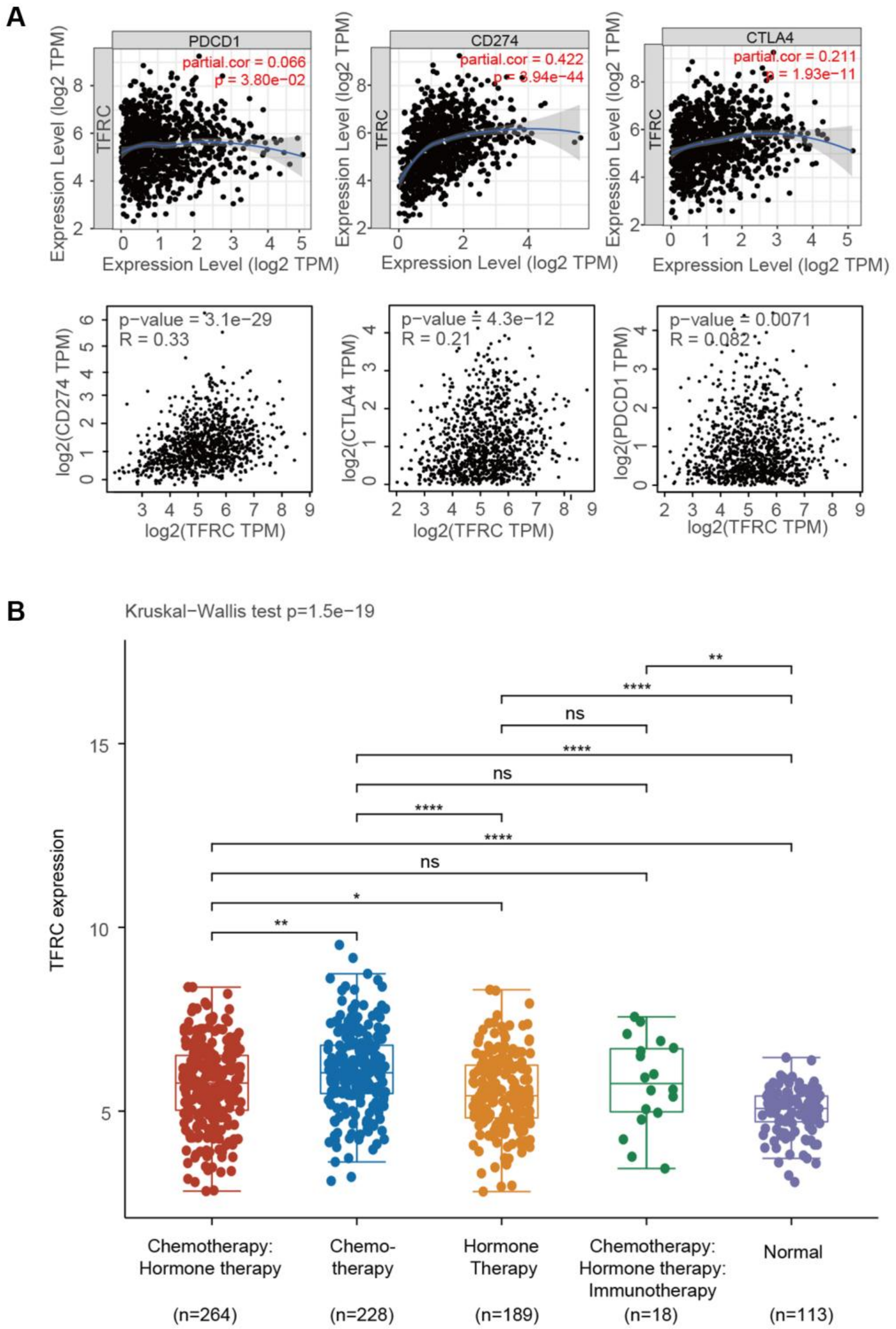

Supplementary Figure 5. (**A**) Correlations between TfR1 and CTLA-4, PD-L1, and PD-1 expression in BC according to the TIMER (upper) and GEPIA (lower) databases. (**B**) TfR1 expression in BC patients with different treatments was investigated based on the TCGA dataset using the R software package ggplot2 with the Kruskal-Wallis test. *< 0.05, **< 0.01, ***< 0.001, ****< 0.0001.
